# Continuous 24-hour measurement of intraocular pressure in millimeters of mercury (mmHg) using a novel contact lens sensor: Comparison with pneumatonometry

**DOI:** 10.1371/journal.pone.0248211

**Published:** 2021-03-23

**Authors:** Kevin Gillmann, Robert Wasilewicz, Kirsten Hoskens, Sonja Simon-Zoula, Kaweh Mansouri

**Affiliations:** 1 Glaucoma Research Centre, Montchoisi Clinic, Swiss Visio Network, Lausanne, Switzerland; 2 [WASILEWICZ]-Eye Clinic, Poznan, Poland; 3 Sensimed AG, Lausanne, Switzerland; 4 Department of Ophthalmology, University of Colorado School of Medicine, Aurora, Colorado, United States of America; Xiamen University, CHINA

## Abstract

**Purpose:**

To address the unmet need of continuous IOP monitoring, a Pressure-Measuring Contact Lens (PMCL) was developed to measure IOP in millimeters of mercury (mmHg) continuously over 24 hours. The present study assessed the reliability of the novel PMCL.

**Methods:**

In this prospective open-label clinical study, healthy and open-angle glaucoma (OAG) subjects were fitted with the PMCL, and pneumatonometry was performed on study eyes (in absence of the PMCL) and on fellow eyes before, during, and after provocative tests. The primary outcome measures were (1) mean IOP difference between same-eye measurements, and (2) percentage of timepoints at which IOP measured by the PMCL was within 5 mmHg of that measured by pneumatonometry in the fellow eye.

**Results:**

Eight subjects were analysed (4 healthy, 4 OAG). The average difference in successive IOP measurements made by pneumatonometry and with the PMCL was 2.0±4.3mmHg at placement-time, and 6.5±15.2mmHg at removal time. During water drinking test, a significant increase in IOP was detected both by PMCL in the study eye (2.4±2.5mmHg, p = 0.03) and by pneumatonometry in the fellow eye (1.9±1.9mmHg, p = 0.02). Over the 24-hour recording, 88.0% of IOP variations measured by the PMCL were within 5mmHg of that measured with the pneumatonometer in the fellow eye. A transient corneal erosion of severe intensity was observed following removal of the PMCL on one single eye, and may have affected measurement accuracy in that eye.

**Conclusions:**

This study is a proof-of-concept for this novel PMCL, and its results are encouraging, with a fair accuracy in IOP values measurement and good sensitivity to subtle IOP variations.

## Introduction

While the exact pathophysiological mechanisms of glaucoma still are not fully understood, intraocular pressure (IOP) remains the only treatable risk factor for the onset and progression of the disease [1]. IOP measurement is one of the most frequently performed test in clinical ophthalmology. However, despite of the ever-growing number of IOP-lowering medications and procedures [2], innovation has been slower when it comes to IOP-measurement techniques, and the gold standard technique has essentially remained the same since 1950, when Goldmann tonometry (GAT) was introduced [3]. Yet, this technique is widely regarded as imperfect and studies have pointed out its flaws both in terms of design and concept [4–7]. Not only is GAT relatively subjective, but the instant nature of standard tonometry techniques also fails to reflect the complexity of real-life IOP variations. Indeed, far from being static, IOP fluctuates widely over the course of 24 hours and through the year, making clinicians’ attempts at describing it with a handful of individual values imprecise at best [8–12]. Over the last decades, several studies have suggested that capturing the dynamic nature of IOP through its physiological and environmentally-induced fluctuations could be key to assessing glaucoma stability [13–15]. Indeed, such variations, including nocturnal pressure spikes, could contribute directly to retinal ganglion cell damage, regardless of IOP values. The currently accepted method to assess short-term fluctuations in a clinical setting relies on diurnal or 24-hour tension curves. During these examinations, tonometry measurements are repeated every single or few hours. Such procedures, however, are cumbersome (consumes scarce resources), expensive (usually requires hospitalization), inconvenient (disturbed sleep cycle as patient is awoken for nocturnal measurements) and may not adequately reflect the IOP fluctuations occurring in real-life activities [16–19].

This unmet need has led to the development of newer devices designed to allow continuous IOP monitoring. Recently, two telemetry devices have become commercially available to give clinicians a glimpse of their patients’ real-life IOP fluctuations. One, a permanent intraocular sensor (EYEMATE, ImplanData, Hannover, Germany), is being assessed in the ARGOS study, with first reports showing similar telemetric IOP profiles compared to those of GAT [20, 21]. Yet, despite encouraging results, its implantable nature reserves it for patients in more advanced stages of the disease. The other commercially available IOP telemetry device, the SENSIMED Triggerfish (Sensimed, Lausanne, Switzerland), is another contact lens sensor (CLS) designed for 24-hour continuous wear. It has to be specifically fitted for each patient, with 3 base curves available, and is well-tolerated, with a mean tolerability visual analogue score (VAS) of 24.3 mm [22]. It accurately detects relative IOP fluctuations with minimal noise, but only reports ocular volume changes related to IOP in arbitrary unit (mVeq) [23, 24]. A recent study by De Moraes et al. has shown significant association between some features of IOP-related CLS profiles and the rate of visual field progression in treated glaucoma patients [25]. While these specificities made it valuable diagnostic tool to confirm a clinical intuition, such as identifying a nocturnal IOP rise or intermittent spikes, its clinical interpretation needs to be improved.

In this context, a novel CLS-based device (Pressure-Measuring Contact Lens [PMCL], Sensimed, Lausanne, Switzerland) was developed to monitor 24-hour IOP in ambulatory conditions, regardless of patients’ positions or activities, including sleep periods. Contrary to the previously developed SENSIMED Triggerfish, that could only assess IOP-related fluctuations in an arbitrary unit, the PMCL was designed to measure actual IOP, in millimeters of mercury (mmHg), continuously over 24 hours. The system is composed of a PMCL that transmits its measurements wirelessly to a periorbital patched adhesive antenna and a recorder. At the end of the recording period, patients return to the clinic for removal of the PMCL, and analysis of the 24-hour IOP measurements stored on the recorder.

A recent article showed comparable values between IOP measured by GAT in one eye and the values acquired by PMCL on the same eye [26]. Furthermore, PMCL was shown to be able to detect IOP variations including those due to a water drinking test (WDT), paralleling the results acquired in the fellow eye using dynamic contour tonometry (DCT). In the present study we assessed the reliability of the novel PMCL for IOP measurements against pneumatonometry, in healthy and glaucomatous subjects exposed to provocative tests including WDT and change in body position.

## Materials and methods

This was an open-label prospective clinical study, conducted at a single site in Poznan, Poland. The study was reviewed and approved by the local ethics committee (IRB; Bioethics Committee of the Wielkopolska Chamber of Medicine, Poznan, Poland) and written informed consent was obtained from all subjects. The study was initially approved by the ethics committee in January 2018. An amended version was approved in May 2018. The study lasted 6 weeks, with the first subject entering on the 23rd of May 2018 and the last subject exiting the study on the 4th of July 2018.

The study was conducted in full compliance with the Declaration of Helsinki and registered at ClinicalTrials.gov (identifier NCT03689088). The study was registered in May 2018, before starting patient recruitment. However, for confidentiality reason related to the breakthrough device, the decision was taken to release the registration later. The authors confirm that all ongoing and related trials for this device are registered.

### Inclusion and exclusion criteria

Healthy subjects and subjects with open-angle glaucoma (OAG) were prospectively enrolled at the investigation site between May and July 2018. Healthy subjects were required to present no structural or functional defect as confirmed by optical coherence tomography (OCT) imaging or biomicroscopic examination of the optic nerve and visual field testing (VF) respectively. Additionally, all of their measured IOP had to be < 22 mmHg. Subjects with OAG were required to have a formal OAG diagnosis based on typical glaucomatous VF defects in keeping with structural defects observed via OCT imaging or biomicroscopic examination of the optic nerve.

In addition, all subjects had to be 18 or older at the time of recruitment, with a body mass index (BMI) ≤ 30 kg/m^2^, a central corneal radius (CCR) between 7.5 mm (45 D) and 7.9 mm (42.75 D), a central corneal thickness (CCT) between 500 μm and 600 μm in both eyes and a good IOP symmetry between eyes to enable inter-eye comparisons. Subjects with a difference in IOP absolute value between eyes of more than 2.5 mmHg in sitting position, and subjects with closed iridocorneal angles on gonioscopic examination, were not included in the study. Furthermore, glaucoma subjects had to be either untreated or “washed-out” from any glaucoma medication for at least four weeks prior to the first measures.

Subjects were excluded from the study if they were diagnosed with any other ocular pathology, if they had previous glaucoma, cataract or refractive surgery, or if they presented any contraindication to contact lens wear or provocative tests.

### Procedures

All enrolled subjects underwent a complete screening examination to confirm their compliance with the inclusion criteria. On the first study day, slit lamp examination was repeated and IOP was measured in both eyes, in sitting position, successively using a Goldmann tonometer and a pneumatonometer. Subjects were then fitted with the PMCL, and pneumatonometer measurements of IOP were repeated in the fellow eye (1) in sitting position, (2) in supine position, (3) 30 minutes before and over 1 hour after a water drinking test (WDT) during which patients were asked to drink 1L of water within 5 minutes. Following the WDT, the recording continued in ambulatory conditions and the subjects resumed their daily activities. They returned after 24 hours for removal of the PMCL. Pneumatonometer measurements in the fellow eye were repeated every 15 min for 1h before the end of the session, then in both eyes immediately after removal of the lens, and slit lamp examination of the eye was performed again. Two consecutive pneumatonometer measurements were made at each of the 25 scheduled timepoint and their results were averaged.

An assessment of subjects’ perceived discomfort was carried out in the form of a visual analogue scale (VAS), both before lens placement and before lens removal. The results were reported in mm as measured on the VAS, from 0 mm (no discomfort) to 100 mm (severe discomfort).

Adverse effects and device deficiencies were recorded throughout the study.

### Device

The PMCL is a non-implantable, temporary CLS intended for continuous recording of IOP for up to 24 hours. The entire setup consists of a PMCL, an adhesive periocular antenna and a recording system. The PMCL is a 15-mm contact lens molded from medical grade silicone elastomers with surfaces treated with oxygen plasma to ensure hydrophilicity and allow overnight wear. In its center, a Micro-Electro-Mechanical System (MEMS) pressure sensor, a circular antenna and a telemetry microprocessor are embedded. The proprietary design of the lens adopts a specific geometry that holds it in place and relaxes the cornea to allow IOP measurements by individually calibrated MEMS sensor. Measurements are acquired continuously at a rate of 1 Hz during the entire duration of the recording, and are supplemented by intense sampling bursts, at 51.2Hz, for 10 seconds every 3 minutes. Readings are digitalized by the built-in microprocessor and sent wirelessly through the periorbital antenna connected to a portable recorder. Upon completion, the recording is transmitted to a computer for visualization.

The individually calibrated pressure sensor embedded at the center of the lens allows for IOP measurement in mmHg in a comparable way to a tonometer. This design drastically differs from the manufacturer’s previous device, the Triggerfish, which consists of a 14.1-mm diameter lens embedding a strain gauge sensitive to changes in ocular dimension measured at the cornea scleral junction.

A single base curve device was available at the time this study was conducted.

### Outcome measures

The primary outcome measures of this study were:

1. the mean IOP difference between the first PMCL measurements and the pre-CLS placement pneumatonometer measurements, and the last PMCL measurements and the post-CLS removal pneumatonometer measurements, respectively, in the study eye,
2. the percentage of timepoints at which variations of IOP measured by the PMCL was within 5 mmHg of that measured by pneumatonometry in the fellow eye, with a threshold for success set at 80%.

The first outcome measure intended to confirm the capabilities of the device to measure IOP values in mmHg, in a comparable way to tonometry, while the second outcome measure intended to validate the capabilities of the device to measure IOP variations. The 5 mmHg window was defined in agreement with ISO 8612:2009 and ANSI Z80.10–2018 norms for Tonometers.

### Statistical analysis

Sample size for this study was based on the hypothesis that IOP measurements with PMCL are within 5 mmHg of the tonometer measurements at more than 80% of the measurement timepoints. With 25 scheduled timepoints over the 24-hour period, 80% of this represents 20 timepoints. The alpha error for the sample size calculation was therefore adjusted for 20 comparisons using the Bonferroni correction and was set at 0.25%. With a standard deviation for PMCL measurements assumed to be 2.5 mmHg, and considering a dropout rate of 10–20%, a sample size of 10 eligible subjects would result in a power of 80% to detect an equivalence margin of 5 mmHg between PMCL and tonometry.

To be considered reliable, PMCL measurements had to be available for at least 80% of the recording. The median of the first burst was compared with IOP measured right before CLS placement, and the median of the last burst was compared with IOP measured right after CLS removal. Similarly, IOP measured in the fellow eye during the recording period were compared with the median of the closest burst. To compare IOP variations regardless of difference in IOP levels between fellow eyes, the overall mean value of PMCL and pneumatonometer was subtracted from individual PMCL and pneumatonometer measurements, respectively. Bland-Altman plots were used to illustrate the relationship between PMCL and pneumatonometer measurements. All statistical analyses were performed with R v.3.5.1 using one or two-sided tests with 95% confidence intervals (CIs) for estimates of performance.

## Results

**Fig 1:**
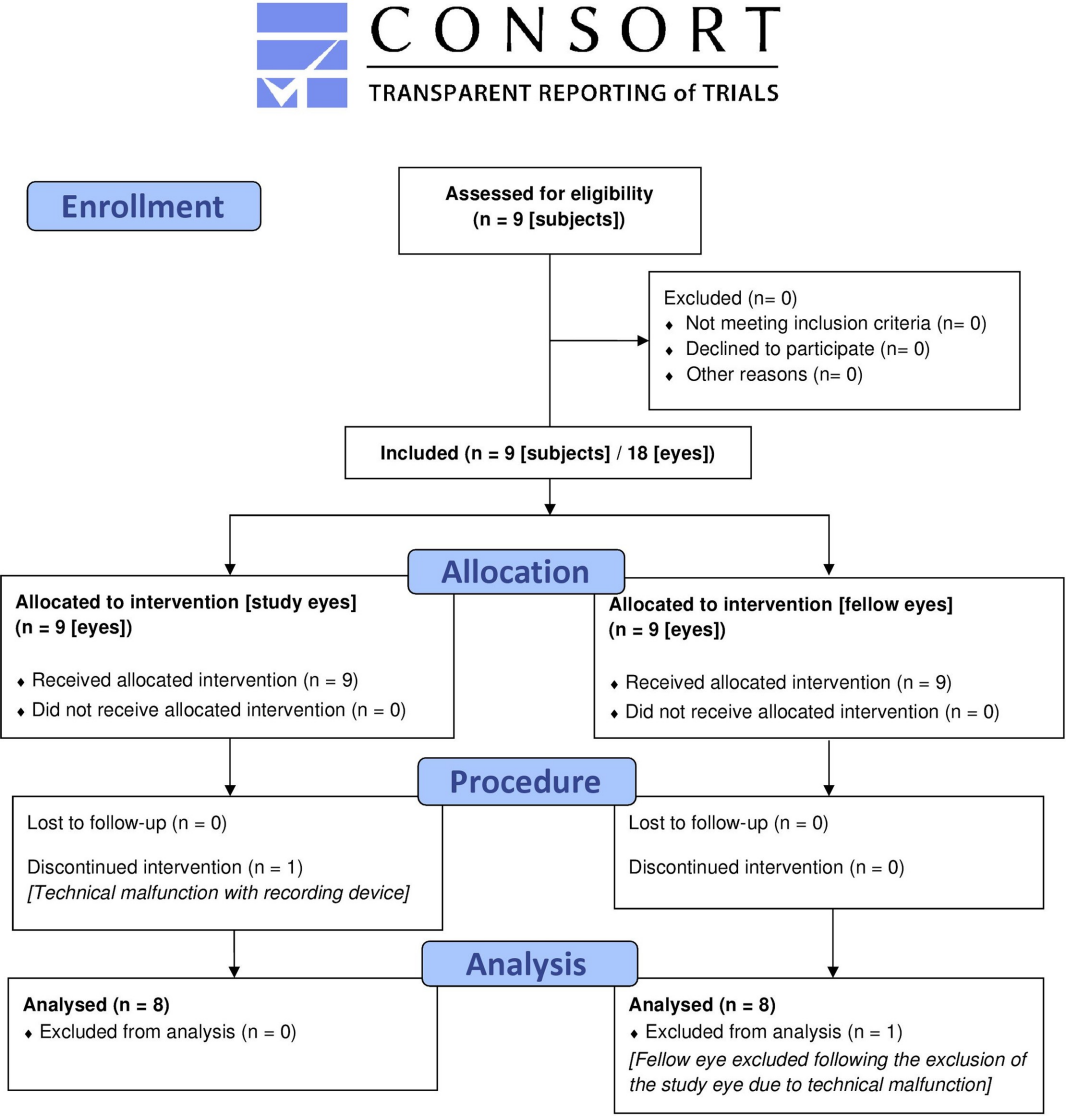
CONSORT flowchart showing the number of patients enrolled, analysed and excluded from analysis.

In total, 9 subjects were enrolled in the study. One subject was excluded from all performance analyses due to a technical malfunction of the PMCL. The performance analyses were therefore carried out on the data of 8 subjects: 4 healthy subjects, 2 with a diagnosis of primary OAG (POAG) and 2 with normal tension glaucoma (NTG). Mean age was 52.9 ± 17.2 years, with the glaucoma group being older than healthy subjects (63.0 ± 18.2 vs. 42.7 ± 9.4 years respectively). Fig 1 presents the CONSORT flowchart of subject inclusion.

Table 1 presents the demographics and baseline characteristics of all subjects.

**Table 1:** Demographics and baseline characteristics of all subjects.

			Healthy subjects	POAG	NTG	Glaucoma	All
Count (%)	Diagnosis		4 (50)	2 (25)	2 (25)	4 (50)	8 (100)
	Gender	F	3 (60)	0 (0)	2 (40)	2 (40)	5 (62.5)
		M	1 (33.3)	2 (66.6)	0 (0)	2 (66.6)	3 (37.5)
	Study eye	L	2 (66.6)	0 (0)	1 (33.3)	1 (33.3)	3 (37.5)
		R	2 (40)	2 (40)	1 (20)	3 (60)	5 (62.5)
Mean ± SD	Age (y)		42.7 ± 9.4	48.5 ± 12.0	77.5 ± 2.12	63.0 ± 18.2	52.9 ± 17.2
	BMI (kg/m^2^)		24.0 ± 7.3	27.0 ± 1.4	26.0 ± 5.6	26.5 ± 3.4	25.2 ± 5.5
	VF MD (dB)		-0.2 ± 0.8	0.3 ± 1.1	-7.3 ± 8.3	-3.5 ± 6.5	-1.8 ± 4.7
	OCT RNFL (μm)		97.7 ± 20.3	77.5 ± 17.5	76.0 ± 17.7	76.7 ± 11.3	87.2 ± 18.9
	Axial length (mm)		23.8 ± 0.4	24.6 ± 0.5	22.7 ± 0.0	23.6 ± 1.1	23.7 ± 0.8
	Refraction (D)	Sph	-0.9 ± 1.3	0.2 ± 0.9	1.1 ± 0.0	0.7 ± 0.7	-0.1 ± 1.3
		Cyl	-1.1 ± 1.3	-0.4 ± 0.1	-1.2 ± 0.8	-0.8 ± 0.6	-1.0 ± 1.0
	corneal epithelial thickness (μm)		50.2 ±2.9	47.5 ± 0.7	41.5 ± 0.7	44.5 ± 3.5	47.4 ± 4.3
	CCT (μm)		541.7 ± 12.5	549.0 ± 22.6	518.5 ± 12.0	533.7 ± 23.0	537.7 ± 17.7
	CCR (mm)		7.6 ± 0.1	7.9 ± 0.0	7.5 ± 0.0	7.7 ± 0.2	7.7 ± 0.1
	Corneal biomechanics	CH	12.2 ± 1.3	9.0 ± 1.9	10.1 ± 1.5	9.6 ± 1.5	10.9 ± 1.9
		CRF	12.5 ± 1.8	13.5 ± 0.3	9.8 ± 0.9	11.7 ±2.2	12.1 ± 1.9
		IOP_cc_ (mmHg)	15.5 ± 3.8	29.7 ± 7.6	16.3 ± 3.8	23.0 ± 9.1	19.2 ± 7.6*
		IOP_g_ (mmHg)	17.2 ± 4.2	29.7 ± 6.4	15.4 ±2.3	22.5 ± 9.2	19.9 ± 7.2*
	IOP_GAT_ (mmHg)		15.8 ± 3.9	24.5 ± 2.1	17.0 ± 0.0	20.7 ± 4.5	18.3 ± 4.7*
	IOP_Pneumatonometer_ (mmHg)		20.5 ± 4.2	29.4 ± 7.2	21.8 ± 0.0	25.6 ±6.1	23.0 ± 5.6*

*p-value resulting from a Kruskal-Wallis test did not show any significant difference between tonometers.

SD: standard deviation; POAG: Primary open angle glaucoma; NTG: Normal tension glaucoma; F: female; M: male; L: left; R: right; y: years; VF MD: visual field mean deviation; dB: decibel; OCT RNFL: Optical coherence tomography retina nerve fiber layer; Sph: spherical; Cyl: cylinder; CCT: central corneal thickness; CCR: central corneal radius; CH: corneal hysteresis; CRF: corneal resistance factor.

### Intraocular pressure values

Mean IOP measured by pneumatonometry in the study eye prior to PMCL placement was 20.5 ± 4.2 and 25.6 ± 6.1 in healthy subjects and glaucoma patients, respectively, leading to an average IOP of 23.0 ± 5.6 mmHg (Table 1). The mean of the first PMCL measurements following placement, defined as the median value of the first burst sampling, was 21.0 ± 2.6 mmHg for all the subjects, 20.6 ± 3.9 mmHg in healthy subjects and 21.4 ± 1.1 mmHg in glaucoma patients. As a comparison the IOP values measured by GAT were 15.8 ± 3.9 mmHg in healthy subjects and 20.7 ±4.5 mmHg in glaucoma patients with a mean IOP of 18.3 ± 4.7 for all the participants.

Following PMCL removal, the mean IOP measured by pneumatonometry in the study eye was 22.2 ± 5.8 mmHg (20.3 ± 5.4 mmHg and 24.1 ± 6.4 mmHg, for healthy subjects and glaucoma patients, respectively). The mean of the last PMCL measurements before removal (including the subject with the severe corneal erosion) was 15.7 ± 10.5 mmHg. This represents an average difference, in absolute value, of 6.5 ± 15.2 mmHg.

**Fig 2:**
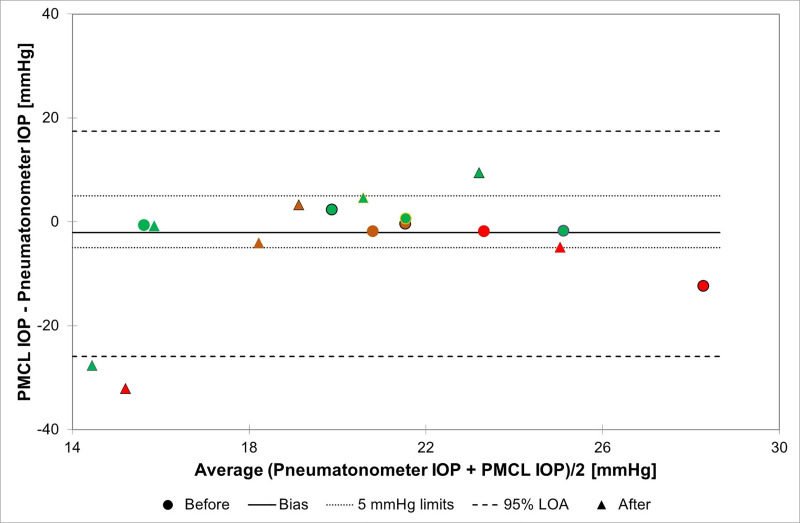
Bland-Altman plot illustrating the relationship between PMCL and pneumatonometry IOP measurements acquired on the same eye before and after 24h recording with PMCL. Each "before" marker (circle) is associated to an "after" marker (triangle) circled with the same colour. Different colours and outlines are used for each subject: green for healthy subjects; red and brown for glaucoma patients. (PMCL: pressure-measuring contact lens; IOP: Intraocular pressure; LOA: Limits of agreement).

Out of these 16 same-eye comparisons, 12 PMCL measurements were within 5 mmHg of that of the pneumatonometer (75%; 87.5% at the start of the recording, 62.5% at the end) (Fig 2).

### Intraocular pressure variations

#### 24h IOP variations

**Fig 3:**
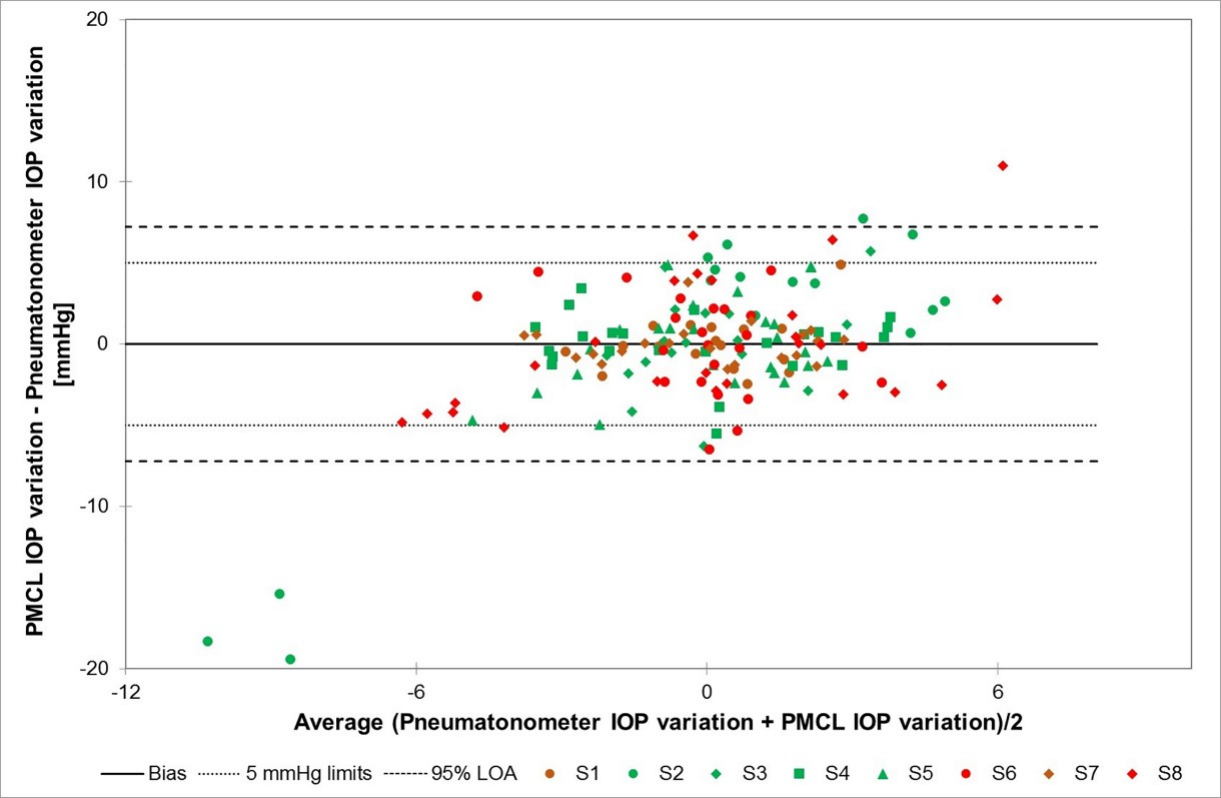
Bland-Altman plot illustrating the relationship between PMCL and pneumatonometry IOP variations in the fellow eyes of each subject through all measurement timepoints. Different colours (green for healthy subjects; red and brown for glaucoma patients) and shapes are used for each subject (S1-S8). (PMCL: pressure-measuring contact lens; IOP: Intraocular pressure; LOA: Limits of agreement).

Out of all the timepoints over the 24-hour recording, 88.0% of IOP variations measured by the PMCL in the study eye were within 5 mmHg of that measured with the pneumatonometer in the fellow eye (95% confidence interval: 74.1%-100%). Fig 3 presents a Bland-Altman plot illustrating the relationship between PMCL and pneumatonometry IOP variations at each measurement timepoint. Despite some outliers, most of points are within the ± 5 mmHg range.

#### Provocative test variations: WDT

**Fig 4:**
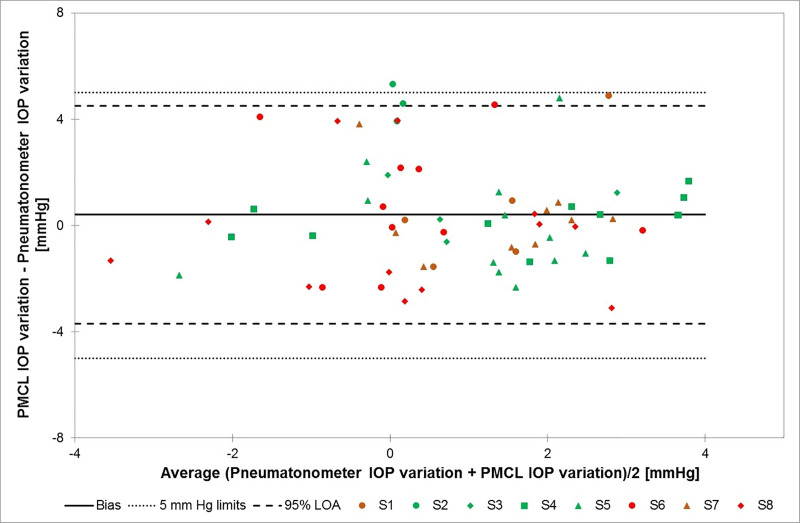
Bland-Altman plot illustrating the relationship between PMCL and pneumatonometry IOP variations in the fellow eyes of each subject during the water-drinking test. Different colours (green for healthy subjects; red and brown for glaucoma patients) and shapes are used for each subject (S1-S8). (PMCL: pressure-measuring contact lens; IOP: Intraocular pressure; LOA: Limits of agreement).

Mean IOP recorded with the pneumatonometer in the fellow eyes 30 minutes before the start of the WDT was 22.4± 6.0 mmHg. At the end of this provocative test, mean IOP was 24.3 ± 6.0 mmHg, showing a mean significant IOP increase of 1.9 ± 1.9 mmHg (p = 0.02) as a result of the WDT. In comparison, the mean IOP recorded by the PMCL in the study eyes prior to the start of the WDT was 20.1 ± 4.2 mmHg. At the end of the provocative test, mean IOP was 22.6 ± 4.9 mmHg (mean significant increase of 2.4 ± 2.5 mmHg, p-value = 0.03). Fig 4 presents a Bland-Altman plot illustrating the relationship between PMCL and pneumatonometry IOP variations during WDT. Despite some outliers, most of points are within the ± 5 mmHg range.

#### Provocative test variations: Body posture

Mean IOP recorded with the pneumatonometer in the fellow eyes when subjects were in supine position for 40 min was 26.6 ± 7.4 mmHg (23.3 ± 2.3 mmHg and 30.0 ± 9.5 mmHg, for healthy subjects and glaucoma patients, respectively). When subjects went back to sitting position mean IOP significantly decreased to 22.3 ± 6.3 mmHg (p = 0.001), showing the same range of decrease amplitude for healthy subjects and glaucoma patients). In comparison, mean IOP recorded with the PMCL in the study eyes while the subjects were in supine position was 20.8 ± 3.7 (with no statistical difference between healthy subjects and glaucoma patients) slightly decreasing to 20.0 ± 3.7 mmHg in sitting position with a p-value of 0.045, showing slightly higher decrease amplitude for glaucoma patients.

### Tolerability

Wearing the PMCL was associated with some degree of discomfort in all the 9 subjects included in the study, with a mean VAS discomfort score of 12.6 ± 19.5 before PMCL placement, increasing to 64.2 ± 28.6 before lens removal, resulting in a mean score of 51.7 ± 29.0 for the overall discomfort (50.0 ± 31.2 and 53.8 ± 30.6, for healthy subjects and glaucoma patients, respectively). A transient corneal erosion of severe intensity was observed following removal of the PMCL on one single eye.

## Discussion

These preliminary results confirmed that the PMCL is able to measure IOP continuously for up to 24 hours, providing a large number of measurements during daytime and sleep period. Across all timepoints, 88.0% of PMCL measurements were within 5 mmHg of that made with a pneumatonometer on the fellow eyes. The ± 5 mmHg threshold being the generally accepted limit for testing new tonometers, this suggests that the PMCL is capable of accurately measuring IOP variations. In terms of sensitivity, the PMCL was able to detect IOP fluctuations of relatively small amplitudes, such as that induced by a WDT. In the present study, the PMCL detected a mean increase of 2.4 ± 2.5 mmHg following this provocative test, while IOPs measured by pneumatonometry increased on average by 1.9 ± 1.9 mmHg. In terms of IOP values in the study eye, we reported an 87.5% accuracy in the pre-PMCL placement measurement. This result is above the success threshold for IOP measurement. Twenty-four hours later, however, the post-PMCL removal IOP measurements was down to 62.5% of values within 5 mmHg of controls. In fact, individual IOP plots show a trend of reduced accuracy on most of the timepoints on the second day of continuous PMCL wear. While the reasons for this reduction in accuracy around the end of the recording period are not completely clear, it could be suspected that the severe corneal erosion of one subject eye resulting in corneal epithelial modifications may have affected the PMCL measurements. These changes in the eye structure might have reduced the capacity of the PMCL to adopt the specific geometry that hold it in place and relaxes the cornea to allow for IOP measurements. One should also consider the impact of the repeated pneumatonometry measurements on the cornea, reducing the reliability of the tonometer measurements. Indeed, the present study used a pneumatonometer to assess the efficacy and accuracy of the PMCL in the measurement of IOP values and IOP variations. The choice of a pneumatonometer was guided by its reliability in various body positions, allowing to test for IOP variations with postural changes, and by the reduced impact of corneal thickness on pneumatonometry measurements compared to GAT [27]. However, repeated pneumatonometry measurements in short intervals may affect IOP in the control eye, widening the discrepancy with the study eye.

In comparison with the present results, a study by Wong et al. comparing GAT with two widely used tonometers (the Tonopen XL and the iCare ic100 rebound tonometer) reported 86.5% and 78.4% of measurements within the 5 mmHg threshold, respectively [28]. With regards to device sensitivity, interestingly, the reported effect of a WDT on IOP in the literature was closer to that detected by the PMCL (2.4 mmHg) compared to pneumatonometry (1.9 mmHg), with observed pressure increases in the order of 3.5 mmHg [29]. The PMCL also detected subtle IOP increases associated with a change in body position from supine to sitting (0.8 ± 0.9 mmHg), which corresponds to the amplitude of IOP changes observed amongst the general population (0.8 ± 1.3 mmHg) [30]. Interestingly, over the same postural change, pneumatonometry detected a mean IOP change of 3.8 mmHg. The difference in magnitude observed between PMCL and pneumatonometry variations in different body positions may constitute a chance finding which might be explained by the relatively small sample size, by the fact that these measurements were made in controlateral eyes, or by aberrant measurements caused by borderline keratometries. Indeed, only a single base curve PMCL was available at the time of the study. All the subjects met the keratometry criteria for inclusion in the study; however, two of them showed borderline keratometries. Data corresponding to these two patients represent most of the outlying data points. Furthermore, it cannot be excluded that some true IOP changes may remain undetected by the PMCL.

This study has several limitations. The main one is its small sample size. The second is the method of comparison involving the averaging of 2 successive pneumatonometer IOP measurements and its comparison with the median of all PMCL measurements acquired during a sampling burst following the lens placement. However, no alternative could be found to assess the capability of the lens to measure IOP values as no tonometry measurement can be performed when the lens is on the eye, and IOP values notoriously differ between fellow eyes. This has also led the investigators to consider the mere amplitudes of IOP variations to assess the ability of the lens to detect IOP variations in the contralateral eye, as these were shown to be relatively similar between both eyes, even in asymmetric glaucoma [31].

## Conclusions

This study is a proof-of-concept for this novel PMCL, and its results are encouraging, with a fair accuracy in IOP values measurement compared to pneumatonometry, and a good sensitivity to subtle IOP variations over 24 hours. However, the device still needs to be optimized to improve performance, safety and tolerability, and tested in larger cohorts. Finally, as the role of IOP fluctuations in the pathophysiology of glaucoma become better understood, the P19MCL could offers a readily-available and non-invasive way to detect these characteristics and improve glaucoma management. Cost-effectiveness of 24-hour IOP monitoring technologies will need to be assessed in comparison with more traditional methods.

## Supporting information

S1 Checklist (PDF)

S1 Table Baseline characteristics of all study subjects. (DOCX)

S2 Table Pre-PMCL fitting (SD0) and post-PMCL removal (SD1) comparison between PMCL and pneumatonometer intraocular pressure in the study eye. (DOCX)

S3 Table Comparison between study eye and fellow eye pneumatonometer intraocular pressure at baseline. (DOCX)

S4 Table Comparison between study eye PMCL and fellow eye pneumatonometer intraocular pressure variations during study procedures. (DOCX)

S5 Table VAS scores before and after PMCL wear. (DOCX)

S1 File (PDF)
