# Supplementary material for: Continuous 24-hour measurement of intraocular pressure in millimeters of mercury (mmHg) using a novel contact lens sensor: Comparison with pneumatonometry
Source: PLoS One. 2021 Mar 23;16(3):e0248211. doi: 10.1371/journal.pone.0248211 (PMC7987168; doi:10.1371/journal.pone.0248211)
Supplement: S1 Table — (DOCX) [file pone.0248211.s002.docx]

**S1 Table.** Baseline characteristics of all study subjects.

| Patient Number | Diagnosis | Gender | Study eye | Age | Height | Weight |
| --- | --- | --- | --- | --- | --- | --- |
| 1 | NTG | female | L | 76 | 1.58 | 75 |
| 2 | Healthy subject | male | R | 45 | 1.7 | 98 |
| 3 | Healthy subject | female | R | 37 | 1.75 | 58 |
| 5 | Healthy subject | female | L | 55 | 1.55 | 60 |
| 6 | Healthy subject | female | L | 34 | 1.65 | 49 |
| 7 | POAG | male | R | 40 | 1.82 | 87 |
| 8 | NTG | female | R | 79 | 1.64 | 59 |
| 9 | POAG | male | R | 57 | 1.82 | 91 |
