# Supplementary material for: Continuous 24-hour measurement of intraocular pressure in millimeters of mercury (mmHg) using a novel contact lens sensor: Comparison with pneumatonometry
Source: PLoS One. 2021 Mar 23;16(3):e0248211. doi: 10.1371/journal.pone.0248211 (PMC7987168; doi:10.1371/journal.pone.0248211)
Supplement: S2 Table — (DOCX) [file pone.0248211.s003.docx]

# S2 Table. Pre-PMCL fitting (SD0) and post-PMCL removal (SD1) comparison between PMCL and pneumatonometer intraocular pressure in the study eye.

| Patient Number | Diagnosis | Pneuma SD0 | PMCL SD0 | (Pneuma - PMCL) SD0 | Pneuma SD1 | PMCL SD1 | (Pneuma - PMCL) SD1 |
| --- | --- | --- | --- | --- | --- | --- | --- |
| 1 | NTG | 21.75 | 21.34 | 0.41 | 17.5 | 20.75 | -3.25 |
| 2 | Healthy subject | 26 | 24.24 | 1.76 | 28.25 | 0.6396 | 27.61 |
| 3 | Healthy subject | 21.25 | 21.85 | -0.6 | 18.25 | 22.92 | -4.67 |
| 5 | Healthy subject | 16 | 15.25 | 0.75 | 16.25 | 15.45 | 0.8 |
| 6 | Healthy subject | 18.75 | 21 | -2.25 | 18.5 | 27.91 | -9.41 |
| 7 | POAG | 24.25 | 22.39 | 1.86 | 27.5 | 22.58 | 4.92 |
| 8 | NTG | 21.75 | 19.87 | 1.88 | 20.25 | 16.18 | 4.07 |
| 9 | POAG | 34.5 | 22.08 | 12.42 | 31.25 | -0.85 | 32.1 |
