# Supplementary material for: Continuous 24-hour measurement of intraocular pressure in millimeters of mercury (mmHg) using a novel contact lens sensor: Comparison with pneumatonometry
Source: PLoS One. 2021 Mar 23;16(3):e0248211. doi: 10.1371/journal.pone.0248211 (PMC7987168; doi:10.1371/journal.pone.0248211)
Supplement: S3 Table — (DOCX) [file pone.0248211.s004.docx]

# S3 Table. Comparison between study eye and fellow eye pneumatonometer intraocular pressure at baseline.

| Patient Number | Diagnosis | Pneuma study eye | Pneuma fellow eye | Pneuma study eye – Pneuma fellow eye |
| --- | --- | --- | --- | --- |
| 1 | NTG | 21.75 | 20.5 | 1.25 |
| 2 | Healthy subject | 26 | 25.25 | 0.75 |
| 3 | Healthy subject | 21.25 | 18.75 | 2.5 |
| 5 | Healthy subject | 16 | 17.75 | -1.75 |
| 6 | Healthy subject | 18.75 | 19.25 | -0.5 |
| 7 | POAG | 24.25 | 26.5 | -2.25 |
| 8 | NTG | 21.75 | 20.75 | 1 |
| 9 | POAG | 34.5 | 38.5 | -4 |
