# Supplementary material for: Continuous 24-hour measurement of intraocular pressure in millimeters of mercury (mmHg) using a novel contact lens sensor: Comparison with pneumatonometry
Source: PLoS One. 2021 Mar 23;16(3):e0248211. doi: 10.1371/journal.pone.0248211 (PMC7987168; doi:10.1371/journal.pone.0248211)
Supplement: S4 Table — (DOCX) [file pone.0248211.s005.docx]

# S4 Table. Comparison between study eye PMCL and fellow eye pneumatonometer intraocular pressure variations during study procedures.

| ***Patient Number*** | ***Provocative tests*** | ***Mean Pneuma*** | ***Mean PMCL*** | ***Mean Pneuma - mean*** | ***Mean PMCL - mean*** |
| --- | --- | --- | --- | --- | --- |
| 1 | none | 20.75 | 22.34 | -0.92 | -1.09 |
| 1 | none | 22 | 24.63 | 0.33 | 1.21 |
| 1 | none | 20.75 | 23.69 | -0.92 | 0.26 |
| 1 | none | 20 | 21.63 | -1.67 | -1.80 |
| 1 | none | 21.75 | 22.90 | 0.08 | -0.53 |
| 1 | body posture | 24.25 | 24.23 | 2.58 | 0.80 |
| 1 | none | 20.5 | 20.27 | -1.17 | -3.16 |
| 1 | none | 20.75 | 22.54 | -0.92 | -0.88 |
| 1 | WDT | 23 | 23.20 | 1.33 | -0.23 |
| 1 | WDT | 22.75 | 25.44 | 1.08 | 2.02 |
| 1 | WDT | 22 | 28.64 | 0.33 | 5.22 |
| 1 | WDT | 23.75 | 24.53 | 2.08 | 1.11 |
| 1 | WDT | 21.75 | 23.71 | 0.08 | 0.29 |
| 1 | none | 22 | 23.69 | 0.33 | 0.26 |
| 1 | none | 23.75 | 23.04 | 2.08 | -0.39 |
| 1 | none | 21.25 | 24.04 | -0.42 | 0.61 |
| 1 | none | 20 | 22.89 | -1.67 | -0.53 |
| 1 | none | 19 | 20.26 | -2.67 | -3.16 |
| 2 | none | 26 | 24.47 | 3.63 | 5.72 |
| 2 | none | 22.75 | 22.85 | 0.38 | 4.10 |
| 2 | none | 23.25 | 26.38 | 0.88 | 7.63 |
| 2 | none | 22.25 | 22.43 | -0.13 | 3.68 |
| 2 | none | 26.25 | 23.30 | 3.88 | 4.55 |
| 2 | body posture | 26 | 24.97 | 3.63 | 6.23 |
| 2 | none | 19.75 | 22.23 | -2.63 | 3.48 |
| 2 | none | 21.75 | 25.83 | -0.63 | 7.08 |
| 2 | WDT | 19.75 | 21.43 | -2.63 | 2.69 |
| 2 | WDT | 20.25 | 21.20 | -2.13 | 2.46 |
| 2 | WDT | 20.5 | 20.80 | -1.88 | 2.05 |
| 2 | none | 21 | 21.51 | -1.38 | 2.76 |
| 2 | none | 22.5 | 20.61 | 0.13 | 1.87 |
| 2 | none | 21.25 | -0.73 | -1.13 | -19.47 |
| 2 | none | 21.25 | 2.23 | -1.13 | -16.51 |
| 2 | none | 23.5 | 0.43 | 1.13 | -18.32 |
| 3 | none | 20.5 | 21.85 | 0.53 | -3.61 |
| 3 | none | 19.25 | 22.93 | -0.72 | -2.53 |
| 3 | none | 19.5 | 26.86 | -0.47 | 1.39 |
| 3 | none | 19.5 | 24.47 | -0.47 | -0.99 |
| 3 | none | 23.05 | 22.26 | 3.08 | -3.21 |
| 3 | body posture | 23.5 | 26.11 | 3.53 | 0.65 |
| 3 | none | 18.25 | 23.06 | -1.72 | -2.40 |
| 3 | none | 19.25 | 23.65 | -0.72 | -1.81 |
| 3 | WDT | 22.25 | 28.96 | 2.28 | 3.50 |
| 3 | WDT | 21 | 25.87 | 1.03 | 0.41 |
| 3 | WDT | 19 | 26.38 | -0.97 | 0.92 |
| 3 | WDT | 20.5 | 26.21 | 0.53 | 0.74 |
| 3 | none | 20.5 | 31.69 | 0.53 | 6.23 |
| 3 | none | 19.5 | 25.07 | -0.47 | -0.39 |
| 3 | none | 18.25 | 25.86 | -1.72 | 0.40 |
| 3 | none | 19 | 24.67 | -0.97 | -0.79 |
| 3 | none | 16.75 | 26.96 | -3.22 | 1.50 |
| 5 | none | 15.25 | 15.52 | -2.54 | -3.84 |
| 5 | none | 13.75 | 17.72 | -4.04 | -1.65 |
| 5 | none | 15 | 15.78 | -2.79 | -3.58 |
| 5 | none | 13.75 | 16.34 | -4.04 | -3.02 |
| 5 | none | 20.75 | 16.81 | 2.96 | -2.55 |
| 5 | body posture | 20 | 17.68 | 2.21 | -1.68 |
| 5 | none | 13.5 | 18.48 | -4.29 | -0.88 |
| 5 | none | 14.75 | 15.90 | -3.04 | -3.46 |
| 5 | WDT | 16 | 17.13 | -1.79 | -2.23 |
| 5 | WDT | 15.75 | 17.95 | -2.04 | -1.42 |
| 5 | WDT | 17 | 18.19 | -0.79 | -1.18 |
| 5 | WDT | 19 | 20.64 | 1.21 | 1.27 |
| 5 | WDT | 20.25 | 22.24 | 2.46 | 2.88 |
| 5 | WDT | 21 | 23.61 | 3.21 | 4.25 |
| 5 | WDT | 21.25 | 23.21 | 3.46 | 3.84 |
| 5 | WDT | 21.25 | 23.22 | 3.46 | 3.86 |
| 5 | WDT | 20.75 | 23.98 | 2.96 | 4.62 |
| 5 | WDT | 21.25 | 21.49 | 3.46 | 2.13 |
| 5 | WDT | 19.75 | 22.02 | 1.96 | 2.66 |
| 5 | WDT | 20.25 | 20.45 | 2.46 | 1.09 |
| 5 | none | 19.5 | 21.67 | 1.71 | 2.31 |
| 5 | none | 18 | 19.11 | 0.21 | -0.25 |
| 5 | none | 16.5 | 20.14 | -1.29 | 0.78 |
| 5 | none | 15.5 | 17.75 | -2.29 | -1.61 |
| 5 | none | 15 | 17.04 | -2.79 | -2.33 |
| 6 | none | 20 | 21.09 | -2.24 | -2.56 |
| 6 | none | 19 | 25.28 | -3.24 | 1.62 |
| 6 | none | 20.75 | 23.15 | -1.49 | -0.51 |
| 6 | none | 20 | 22.30 | -2.24 | -1.36 |
| 6 | none | 24 | 23.05 | 1.76 | -0.60 |
| 6 | body posture | 22.5 | 18.96 | 0.26 | -4.70 |
| 6 | none | 19.75 | 16.48 | -2.49 | -7.18 |
| 6 | none | 20.25 | 18.65 | -1.99 | -5.01 |
| 6 | WDT | 20.5 | 20.04 | -1.74 | -3.61 |
| 6 | WDT | 25 | 24.09 | 2.76 | 0.43 |
| 6 | WDT | 25 | 25.08 | 2.76 | 1.42 |
| 6 | WDT | 24.5 | 25.45 | 2.26 | 1.79 |
| 6 | WDT | 24.5 | 24.16 | 2.26 | 0.50 |
| 6 | WDT | 25.25 | 25.62 | 3.01 | 1.96 |
| 6 | WDT | 23.5 | 25.31 | 1.26 | 1.65 |
| 6 | WDT | 24.25 | 24.27 | 2.01 | 0.61 |
| 6 | WDT | 22 | 28.20 | -0.24 | 4.55 |
| 6 | WDT | 23 | 25.67 | 0.76 | 2.01 |
| 6 | WDT | 20.75 | 24.56 | -1.49 | 0.90 |
| 6 | WDT | 21.5 | 23.84 | -0.74 | 0.18 |
| 6 | none | 23 | 23.16 | 0.76 | -0.50 |
| 6 | none | 21 | 23.38 | -1.24 | -0.28 |
| 6 | none | 22.75 | 25.56 | 0.51 | 1.91 |
| 6 | none | 21.25 | 25.90 | -0.99 | 2.25 |
| 6 | none | 22 | 28.18 | -0.24 | 4.52 |
| 7 | none | 30.75 | 22.58 | 3.30 | -3.21 |
| 7 | none | 29.25 | 24.43 | 1.80 | -1.35 |
| 7 | none | 28 | 26.88 | 0.55 | 1.09 |
| 7 | none | 28.25 | 25.29 | 0.80 | -0.49 |
| 7 | none | 30 | 24.94 | 2.55 | -0.84 |
| 7 | body posture | 32.25 | 28.21 | 4.80 | 2.43 |
| 7 | none | 30.75 | 23.74 | 3.30 | -2.04 |
| 7 | none | 26.75 | 24.69 | -0.70 | -1.10 |
| 7 | WDT | 28.5 | 24.50 | 1.05 | -1.29 |
| 7 | WDT | 27.75 | 23.75 | 0.30 | -2.03 |
| 7 | WDT | 26.5 | 29.38 | -0.95 | 3.60 |
| 7 | WDT | 27 | 26.05 | -0.45 | 0.26 |
| 7 | WDT | 27.5 | 25.78 | 0.05 | -0.01 |
| 7 | WDT | 30.75 | 28.90 | 3.30 | 3.12 |
| 7 | WDT | 28.25 | 26.33 | 0.80 | 0.55 |
| 7 | WDT | 26.75 | 27.20 | -0.70 | 1.42 |
| 7 | WDT | 26.5 | 27.00 | -0.95 | 1.22 |
| 7 | WDT | 23.75 | 26.17 | -3.70 | 0.38 |
| 7 | none | 21.75 | 24.52 | -5.70 | -1.27 |
| 7 | none | 21.25 | 22.50 | -6.20 | -3.28 |
| 7 | none | 26 | 25.96 | -1.45 | 0.17 |
| 7 | none | 25.5 | 26.65 | -1.95 | 0.87 |
| 7 | none | 27.5 | 27.56 | 0.05 | 1.78 |
| 8 | none | 23 | 19.76 | 1.20 | -0.07 |
| 8 | none | 20.25 | 17.86 | -1.55 | -1.97 |
| 8 | none | 18 | 16.60 | -3.80 | -3.23 |
| 8 | none | 19.5 | 16.71 | -2.30 | -3.12 |
| 8 | none | 21 | 19.66 | -0.80 | -0.17 |
| 8 | body posture | 24.75 | 21.42 | 2.95 | 1.59 |
| 8 | none | 20.5 | 18.58 | -1.30 | -1.26 |
| 8 | none | 21 | 19.07 | -0.80 | -0.76 |
| 8 | WDT | 19.5 | 21.35 | -2.30 | 1.52 |
| 8 | WDT | 22 | 19.77 | 0.20 | -0.06 |
| 8 | WDT | 23 | 19.49 | 1.20 | -0.35 |
| 8 | WDT | 24.5 | 22.79 | 2.70 | 2.96 |
| 8 | WDT | 23.75 | 20.97 | 1.95 | 1.13 |
| 8 | WDT | 23.5 | 22.11 | 1.70 | 2.27 |
| 8 | WDT | 24 | 22.24 | 2.20 | 2.41 |
| 8 | WDT | 23.5 | 22.41 | 1.70 | 2.57 |
| 8 | WDT | 24 | 21.32 | 2.20 | 1.49 |
| 8 | none | 24 | 22.20 | 2.20 | 2.37 |
| 8 | none | 22 | 21.47 | 0.20 | 1.63 |
| 8 | none | 19.75 | 17.18 | -2.05 | -2.65 |
| 8 | none | 20.25 | 17.04 | -1.55 | -2.80 |
| 8 | none | 17.75 | 16.33 | -4.05 | -3.50 |
| 9 | none | 38 | 22.17 | 0.62 | 11.61 |
| 9 | none | 42 | 17.93 | 4.62 | 7.36 |
| 9 | none | 36.75 | 16.37 | -0.63 | 5.81 |
| 9 | none | 38.25 | 13.21 | 0.87 | 2.64 |
| 9 | none | 42.75 | 12.96 | 5.37 | 2.39 |
| 9 | body posture | 43.5 | 14.15 | 6.12 | 3.59 |
| 9 | none | 33.75 | 13.61 | -3.63 | 3.04 |
| 9 | none | 35 | 12.55 | -2.38 | 1.98 |
| 9 | WDT | 34.75 | 11.86 | -2.63 | 1.29 |
| 9 | WDT | 35.5 | 12.64 | -1.88 | 2.08 |
| 9 | WDT | 39.25 | 12.49 | 1.87 | 1.92 |
| 9 | WDT | 39 | 12.62 | 1.62 | 2.05 |
| 9 | WDT | 39.75 | 12.90 | 2.37 | 2.33 |
| 9 | WDT | 41.75 | 11.83 | 4.37 | 1.26 |
| 9 | WDT | 39 | 9.76 | 1.62 | -0.81 |
| 9 | WDT | 39 | 9.32 | 1.62 | -1.25 |
| 9 | WDT | 38.25 | 9.67 | 0.87 | -0.90 |
| 9 | WDT | 37.5 | 8.38 | 0.12 | -2.18 |
| 9 | WDT | 35 | 8.33 | -2.38 | -2.24 |
| 9 | WDT | 34.5 | 6.35 | -2.88 | -4.21 |
| 9 | none | 35.75 | 3.81 | -1.63 | -6.76 |
| 9 | none | 34 | 3.55 | -3.38 | -7.02 |
| 9 | none | 34.25 | 3.22 | -3.13 | -7.34 |
| 9 | none | 33.75 | 2.64 | -3.63 | -7.92 |
| 9 | none | 33.5 | 1.85 | -3.88 | -8.72 |
