# Supplementary material for: Continuous 24-hour measurement of intraocular pressure in millimeters of mercury (mmHg) using a novel contact lens sensor: Comparison with pneumatonometry
Source: PLoS One. 2021 Mar 23;16(3):e0248211. doi: 10.1371/journal.pone.0248211 (PMC7987168; doi:10.1371/journal.pone.0248211)
Supplement: S5 Table — (DOCX) [file pone.0248211.s006.docx]

# S5 Table. VAS scores before and after PMCL wear.

| Patient Number | Diagnosis | VAS Before | VAS After | VAS After - VAS Before |
| --- | --- | --- | --- | --- |
| 1 | NTG | 1 | 50 | 49 |
| 2 | Healthy subject | 5 | 100 | 95 |
| 3 | Healthy subject | 0 | 47 | 47 |
| 4 | Healthy subject | 7 | 28 | 21 |
| 5 | Healthy subject | 23 | 45 | 22 |
| 6 | Healthy subject | 2 | 67 | 65 |
| 7 | POAG | 2 | 100 | 98 |
| 8 | NTG | 12 | 41 | 29 |
| 9 | POAG | 61 | 100 | 39 |
