# Supplementary material for: Continuous 24-hour measurement of intraocular pressure in millimeters of mercury (mmHg) using a novel contact lens sensor: Comparison with pneumatonometry
Source: PLoS One. 2021 Mar 23;16(3):e0248211. doi: 10.1371/journal.pone.0248211 (PMC7987168; doi:10.1371/journal.pone.0248211)
Supplement: S1 File — (PDF) [file pone.0248211.s007.pdf]

SENSIMED
innovation in medical micro-technology

CLINICAL INVESTIGATION PLAN

**Device:**	SENSIMED Goldfish
**Study Title:**	A prospective pilot study investigating the use of a sensing contact lens-based device for 24-hour monitoring of intraocular pressure in healthy subjects and patients with open angle glaucoma
**Author(s):**	Sonja Simon-Zoula, PhD and Kaweh Mansouri, MD, MPH
**Principal Investigator:**	Robert Wasilewicz, MD<br>[W]-Eye clinic<br>ul. Krańcowa 83<br>61-048 Poznań<br>Poland
**Telephone:**	+ 48 604555172
**e-mail:**	robert.wasilewicz@w-eyeclinic.com

**Sponsor:**	Sensimed AG<br>Route de Chavannes 37<br>1007 Lausanne, Switzerland
**Medical Responsible:**	Kaweh Mansouri, MD
**Telephone:**	+41 21 6219191
**Cell phone:**	+41 79 1767267
**e-mail:**	kaweh_mansouri@sensimed.ch
**Trial Coordinator:**	Sonja Simon-Zoula, PhD
**Telephone:**	+41 21 6219191
**Fax:**	+41 21 6219193
**Cell phone:**	+41 79 5866297
**E-mail:**	sonja_simon-zoula@sensimed.ch

Other Sensimed personnel who may be contacted by study site personnel concerning this study are listed in a separate document, which will be up-dated when necessary.

SPONSOR SIGNATORY APPROVAL

I have read this protocol and I approve the design of this study:

Signature	***Date of signature***

Kaweh Mansouri, MD
Sensimed AG, Lausanne, Switzerland

PRINCIPAL INVESTIGATOR SIGNATORY APPROVAL PAGE

I have read this protocol and I approve the design of this study:

Signature	***Date of signature***

Robert Wasilewicz, MD
[W]-Eye Clinic, Poznan, Poland

TABLE OF CONTENTS

1 INTRODUCTION 12
1.1 BACKGROUND 12
1.2 GLAUCOMA 12
1.2.1 Pathophysiology 12
1.2.2 Classification and Definitions 13
1.2.3 Epidemiology 13
1.2.4 Treatment 14
1.3 SENSIMED GOLDFISH 15
1.3.1 Initial testing with GF 15
1.4 STUDY RATIONALE 16
1.5 GUIDANCE FOR INVESTIGATOR 17
2 OBJECTIVES 18
3 ENDPOINTS 18
3.1 PRIMARY ENDPOINT 18
3.2 SECONDARY ENDPOINTS 18
3.3 EXPLORATORY ENDPOINTS 19
3.4 SAFETY ENDPOINT 19
4 STUDY DESIGN 19
5 STUDY POPULATION 20
5.1 SUBJECTS 20
5.2 ELIGIBILITY CRITERIA 21
5.2.1 Inclusion Criteria (for both eyes where applicable): 21
5.2.2 Exclusion Criteria 21
5.3 ASSIGNMENT TO STUDY GROUPS 22
6 STUDY PROCEDURES AND ASSESSMENTS 22
6.1 OUTLINE OF STUDY PROCEDURES AND ASSESSMENTS 22
6.1.1 Screening 22
6.1.2 SD0 23
6.1.3 SD1 25
6.2 SUBJECT COMPLETION AND WITHDRAWAL 26
6.3 REPLACEMENT POLICY 26
6.4 CONCOMITANT MEDICATIONS AND THERAPIES 27
6.5 OBSERVATION AND MEASUREMENTS 27
7 SENSIMED GOLDFISH 27
7.1 DEVICE DESCRIPTION 27
7.2 MANUFACTURER 28
8 SAFETY 28
8.1 SUMMARY OF OVERALL RISK AND BENEFITS 28
8.1.1 Anticipated Risks 28
8.1.2 Anticipated Clinical Benefits 29
8.2 DEFINITION OF AN ADVERSE EVENT 29
8.3 DEFINITION OF A SAE 29
8.4 ABNORMAL TEST FINDINGS 30
8.5 DEVICE DEFFICIENCIES 30
8.6 SEVERITY GRADING FOR ADVERSE EVENTS 30
8.7 RELATIONSHIP OF AEs TO GF 30

8.8 COLLECTING, RECORDING AND REPORTING OF AEs AND DEVICE DEFICIENCIES 31
9 STATISTICAL CONSIDERATIONS 32
9.1 OVERVIEW 32
9.2 DESIGN CONSIDERATIONS 33
9.3 ANALYSIS POPULATIONS 33
9.3.1 Safety Analysis Populations 33
9.3.2 Performance Analysis Population 33
9.4 SAMPLE SIZE CONSIDERATIONS 33
9.5 ENDPOINTS 33
9.5.1 Safety endpoint 33
9.5.2 Performance endpoints 34
9.6 STATISTICAL ANALYSIS 34
9.6.1 Overview 34
9.6.2 Safety 34
9.6.3 Performance 35
10 STUDY ADMINISTRATION 35
10.1 REGULATORY AND ETHICAL CONSIDERATIONS 35
10.1.1 Regulatory Authority Approval 35
10.1.2 End of the Study 36
10.2 INVESTIGATOR RESPONSIBILITIES 36
10.2.1 Principal Investigator 36
10.2.2 Subject Confidentiality 36
10.3 DATA MANAGEMENT 37
10.4 STUDY MONITORING 37
10.4.1 Monitoring Visits 37
10.4.2 Protocol Deviation 37
10.5 QUALITY ASSURANCE 38
10.6 STUDY OR SITE DISCONTINUATION 38
10.7 RETENTION OF ESSENTIAL STUDY DOCUMENTS 38
10.8 AGREEMENTS AND INSURANCE 39
11 PUBLICATION 39
12 STUDY TIMETABLE 39
13 APPENDIX A 40
STUDY SCHEDULE 40
14 REFERENCES 41

ABBREVIATIONS

AE	Adverse event
ASADE	Anticipated serious adverse device effect
BP	Blood pressure
CCR	Central corneal radius
CCT	Central corneal thickness
CL	Contact lens
CRF	Case report form
DCT	Dynamic contour tonometer
EC	Ethics committee
EGS	European glaucoma society
GAT	Goldmann applanation tonometry
GCP	Good clinical practice
GF	SENSIMED Goldfish
ICF	Informed consent form
ICH	International conference on harmonisation
IOP	Intraocular pressure
IRB	Institutional review board
ISF	Investigator site file
ISO	International standard organization
MEMS	Micro-Electro-Mechanical System
mmHg	Millimeters Mercury (a measure for pressure)
NTG	Normal tension glaucoma
OAG	Open angle glaucoma
OCT	Optical coherence tomography
OPA	Ocular pulse amplitude
ORA	Ocular response analyser
PACG	Primary angle closure glaucoma
POAG	Primary open-angle glaucoma
PT	Provocative test
QoL	Quality of life
RGC	Retinal ganglion cells
RNFL	Retinal nerve fibre layer
SAE	Serious adverse event
SADE	Serious adverse device effect
SD	Study day
TMF	Trial master file
USADE	Unexpected serious adverse device effect
VAS	Visual analogue scale
VF	Visual field
WDT	Water drinking test
WHO	World Health Organisation

STUDY SYNOPSIS

STUDY TITLE: A prospective pilot study investigating the use of a sensing contact lens-based device for 24-hour monitoring of intraocular pressure in healthy subjects and patients with open angle glaucoma

**STUDY NUMBER:** GF-1703	**NAME OF THE DEVICE:** SENSIMED Goldfish

Rationale:

Glaucoma is characterized by irreversible vision loss through the progressive death of optic nerve fibers unless timely diagnosis and adequate treatment are provided. While elevated intraocular pressure (IOP) is no longer part of the definition of glaucoma it remains the sole proven modifiable risk factor for the onset and progression of glaucoma[1]. Medical therapy is aimed at lowering IOP below a clinically determined target level in order to prevent or slow glaucoma progression.

IOP is known to vary with the time of day as well as with daily activities[1]. IOP fluctuations of as much as 4-5 mmHg in healthy individuals and substantially higher in some glaucoma patients have been commonly reported[2, 3]. The current way of assessing nycthemeral IOP fluctuation is to perform repeated discrete tonometry measurements, allowing only snapshot and non-continuous measurements once per hour in the best cases. Furthermore, the procedure is cumbersome, expensive, inconvenient (disturbed sleep cycle as patient is awoken for nocturnal/sleep period measurements) and may not detect crucial IOP values in time. Therefore, assessing IOP continuously over 24 hours is pivotal for the management of glaucoma.
There have been many efforts in the past decades to search for an ambulatory and frequent method to monitor IOP for 24 hours. Many handheld, portable self-monitoring devices have been evaluated[4-6], however they do not address the crucial issue of IOP behaviour during undisturbed sleep. Implantable telemetric devices have been developed for long-term continuous IOP monitoring[7-9]. This approach, however, requires surgical intervention. Moreover, important drawbacks of implantable IOP monitoring devices are the general inability of these devices to provide measurements during sleep period and the potential need for subsequent re-intervention in cases of device failure or malfunction. A variety of non-invasive contact lens (CL)-based technologies for more widely applicable temporary IOP monitoring are also in development[8, 10]. Sensimed AG has developed a new CL-based device intended to measure IOP over 24 hours.

SENSIMED Goldfish (GF) includes a CL molded from medical grade silicone elastomers with oxygen plasma treated surfaces to ensure hydrophilicity. A Micro-Electro-Mechanical System (MEMS) pressure sensor, an antenna and a telemetry microprocessor are embedded in the lens, allowing for continuous IOP recording. The CL is placed on the eye and sends its signals wirelessly to a recorder via a periorbital patched adhesive antenna. Upon completion, the recording is transmitted to a computer for read-out and visualization.

The purpose of this pilot study is to investigate the safety and tolerability of the GF as well as its ability to detect IOP changes in healthy subjects and open angle glaucoma (OAG) patients.

OBJECTIVE:

The objective of this study is to investigate the use of GF for 24-hour IOP monitoring in healthy subjects and OAG patients

ENDPOINTS:

Primary endpoint:

- Changes in IOP assessed by GF following known physiological and induced changes in IOP

Secondary endpoints:

- Percentage of subjects completing 24-hour session with GF
- Wearing discomfort of GF based on visual analogue scale (VAS)

- Evaluation of GF technical performance based on the percentage of valid GF measurements
- Correlation between IOP and ocular pulse amplitude (OPA) assessed by GF in the GF eye and IOP and OPA measured by tonometry in the fellow eye
- Relationship between GF IOP and blood pressure (BP) measurements over 24 hours

Exploratory endpoints:

- Comparison between healthy subjects and glaucoma patients with regard to IOP changes detected by GF
- Detection of body position changes using the accelerometers incorporated in the GF device

Safety endpoint: Adverse events (AEs) and serious adverse events (SAEs) will be collected throughout the duration of the study.

STUDY DESIGN: This is a prospective pilot study to investigate the safety and tolerability of the GF as well as its ability to detect physiological and induced IOP changes in healthy subjects and OAG a patients.

Glaucoma subjects will be selected based on scheduled appointments (consecutive selection) and invited to participate in the study or could be recruited through advertising. The healthy subjects could also be recruited through advertising or among persons accompanying patients to clinic appointments.

After having signed and dated the subject informed consent form, subjects will undergo an initial ophthalmic examination. Subjects presenting average ocular parameter values such as a central corneal radius (CCR; flat meridian) between 7.5 mm (45 D) and 7.9 mm (42.75 D) and a central corneal thickness (CCT) between 500 µm and 600 µm in both eyes will be eligible for the study. Subject will also need to present a good IOP symmetry between eyes, as defined in the inclusion criteria to be qualified for the study. The GF eye will be randomly selected and tested for the GF CL adhesion.

In line with international and American national standard for ophthalmic instruments, to demonstrate the range of IOP values measurable with GF, subjects will be recruited to be evenly allocated to one of the 3 different groups based on their mean IOP value at screening acquired with Goldman applanation tonometry (GAT):

- IOP between 7 and 16 mmHg (group 1)
- IOP between 17 and 22 mmHg (group 2)
- IOP equal or higher than 23 mmHg (group 3).

Glaucoma subjects will be either naïve to IOP-lowering drug treatment or washed out thereof for a 4-week period before the recording session. In case of washed-out patients, IOP will be reassessed before allocation to one of the IOP groups. If allocation is not possible, patient will be withdraw and replaced.

After baseline ophthalmic examination, the VAS will be assessed. GF CL will then be placed on the GF eye for a 24-hour recording session, simultaneous to a 24h BP recording. During the session, each subject will undergo provocative tests (PT) including postural changes and water drinking test (WDT) that are known to induce a change in IOP. IOP will be measured in the fellow eye at specific time points as well as during the PT, using a tonometer. This will allow comparison between fellow eyes as IOP by tonometry is not assessable in the GF eye during recording. As much as possible, the same operator will measure the IOP for a specific subject. Following the PT, the recordings will be continued in ambulatory conditions with the subjects staying calm at home in order to minimize activity-related variation of IOP. Subject activities will be captured in a logbook. Subjects will also be asked to refrain from lying down except during the nocturnal sleep period to avoid uncontrolled variation of IOP. Upon completion of the session, wearing discomfort will be scored on a VAS. GF and BP monitor will be removed and a final ophthalmic examination will be conducted to exclude or adequately address any adverse events and return the logbook to the investigator. This concludes the study for the subjects.

The overall study duration for an eligible subject is limited to 2 days, if no washed out is needed, 4 weeks otherwise.

A sample size of 12 subjects was estimated to evaluate the equivalence between GF and GAT measurements. Therefore, the study has been planned to recruit at least 12 eligible subjects, 6 healthy subjects (4 in group 1 and 2 in group 2) and 6 patients with glaucoma (2 in group 2 and 4 in group 3).

It is assumed that the recruitment of 12 eligible subjects is completed within 5 weeks from the date of initiation.

Hence, the overall study duration from the first subject accrued until last subject out of the study equates to about 6 weeks. A longer recruitment period may be allotted to allow for sufficient subject enrolment.

STUDY POPULATION:

Inclusion criteria (for both eyes where applicable):

1. For OAG subjects,
   a. a clinical diagnosis of primary open angle glaucoma (POAG), including normal tension glaucoma (NTG) with glaucomatous structural defects, glaucomatous visual fields (VF), and open angles on gonioscopy
   b. all known untreated IOP measurements < 22 millimeters of mercury (mmHg) using GAT for NTG
   c. no IOP-lowering treatment; otherwise, a 4-week wash-out period prior to the recording
2. For healthy subjects, no structural defects, normal VFs, IOP ≤ 21 mmHg and open angles on gonioscopy
3. Aged ≥ 18 years, either gender
4. BMI ≤ 30 kg/m^2
5. CCR (flat meridian) between 7.5 mm (45 D) and 7.9 mm (42.75 D)
6. CCT between 500 µm and 600 µm
7. Difference in IOP absolute value between eyes within 2.5 mmHg at screening in sitting position
8. Same direction of IOP variation (positive or negative) for the 2 eyes when moving from sitting to supine positions at screening
9. Spherical refraction within ±6.00 diopters and cylinder refraction within ±3.00 diopters
10. Having given written informed consent, prior to any investigational procedures

Exclusion criteria (for both eyes where applicable):

1. Ocular pathology (other than glaucoma for glaucoma subjects)
2. Previous glaucoma, cataract or refractive surgery
3. Corneal or conjunctival abnormality, precluding contact lens adaptation
4. Severe dry eye syndrome
5. Subjects with allergy to corneal anesthetic
6. Subjects with contraindications for silicone CL wear
7. Subjects with contraindications for WDT (eg., heart, renal problems)
8. Subjects unable or unwilling to comply with the study procedures
9. Participation in other interventional clinical research within the last 4 weeks

DEVICE: GF is a sensing CL-based device intended to monitor IOP continuously over a period of 24 hours.

STUDY SCHEDULE OF PROCEDURES:

Study flowchart	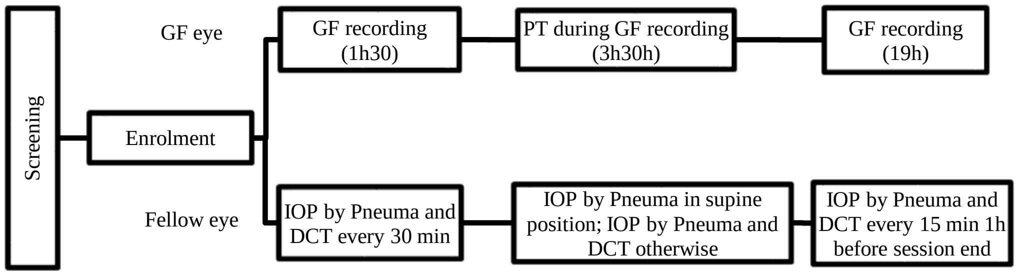
≤ SD0	All screening procedures must be completed at latest on SD0, the day of the GF recording

(screening)	• Demographic data<br>• Medical and ophthalmic history data<br>• Ophthalmic examination (both eyes)<br>▪ VF test (Humphrey 24-2 SITA Standard)<br>▪ Retinal Nerve Fiber Layer (RNFL) thickness by Optical Coherence Tomography (OCT)<br>▪ Corneal epithelial thickness by OCT<br>▪ Gonioscopy<br>▪ Slit lamp examination<br>▪ Refraction test<br>▪ Axial length<br>▪ Topography/keratometry<br>▪ Pachymetry<br>▪ IOP by pneumatonometer in sitting and supine positions<br>▪ Corneal biomechanics by Ocular Response Analyzer (ORA)<br>• Height<br>• Weight<br>• Reassessment of IOP for washed-out OAG patients<br>• Check of all eligibility criteria<br>• GF eye selection (random)<br>• GF CL adhesion testing on the GF eye
SD0	Study procedures will start between 10:30 am and 12 am. GF and BP recordings will be initiated simultaneously.<br>• Ophthalmic examination before GF-CL placing (both eyes)<br>▪ Slit lamp examination (if SD0 different from screening)<br>▪ IOP by GAT in sitting position<br>▪ IOP by dynamic contour tonometer (DCT) in sitting position<br>▪ IOP by Pneumatonometer in sitting position<br>• VAS assessment<br>• Off-eye measurement of the GF CL<br>• GF-CL placing in the GF eye (between 11 am and 12:30 am) and ensure correct fitting<br>• BP monitor placing (between 11 am and 12:30 am)<br>• Following procedures as below (full 24h monitoring):<br>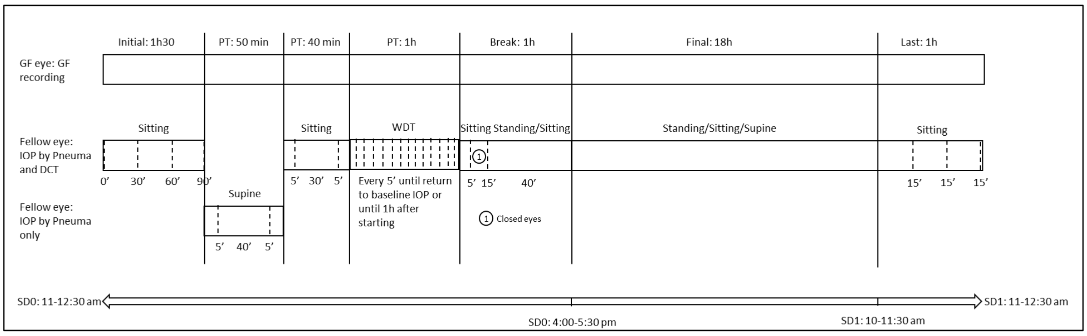

	▪ WDT<br>o Subject will be asked to drink 1.0 L of water within 5 min<br>o IOP in sitting position every 5 min after starting the WDT until return to baseline IOP (± 1 mm Hg) or until 1h after starting the WDT, whichever comes first<br>• GF recording in the GF eye under ambulatory conditions until 24 hours after the start of the recording<br>▪ Patient booklet<br>▪ Subject logbook<br>• Concomitant medication and procedures<br>• AEs and device deficiencies
SD1	Subjects will be requested to arrive at the hospital 1h15 before the end of the recording session.<br>• GF recording in the GF eye, under ambulatory conditions until 24 hours after the start of the recording<br>▪ Subject logbook<br>▪ IOP measurements in the fellow eye as described in the procedure above<br>• BP recording<br>• VAS assessment<br>• GF-CL removal with tweezers 15 min before the end of the recording session<br>• Off-eye measurement of the lens right before the end of the recording session<br>• BP monitor removal at the end of the session<br>• Ophthalmic examination (both eyes)<br>▪ RNFL thickness by OCT<br>▪ Corneal epithelial thickness by OCT<br>▪ Slit lamp examination<br>▪ Axial length<br>▪ Topography/keratometry<br>▪ Pachymetry<br>▪ IOP by GAT in sitting position<br>▪ IOP by DCT in sitting position<br>▪ Corneal biomechanics by ORA<br>• Concomitant medication and procedures<br>• AEs and device deficiencies since last visit<br>• Download of recorded data to the investigator’s computer
**DATA ANALYSIS AND STATISTICS:** The ability of the GF to continuously record known physiological and induced variation in IOP over 24 hours will be assessed and compared to standard tonometry measurements acquired in the fellow eye. Safety, performance and tolerability of the device will be evaluated, as well as relationship of the IOP derived from the device and BP. Comparison between healthy subjects and OAG as well as detection of body position with GF will be explored. Descriptive statistics will be performed on collected data. Correlations and paired comparisons will be presented when appropriate.	

**SAMPLE SIZE AND CONSIDERATIONS:** This is a prospective pilot study to investigate the use of GF for 24-hour IOP monitoring in healthy subjects and patients with OAG. The sample size planned for this study is 12 evaluable subjects (6 healthy subjects and 6 glaucoma patients) that would give relevant statistical significance
**QUALITY STATEMENT:** This study is to be performed in accordance with the protocol, the Declaration of Helsinki, ISO 1455-2011 and all applicable regulatory requirements.

1 INTRODUCTION

1.1 BACKGROUND

Glaucoma is the world's most common degenerative neurological disorder (twice as common as dementia). It is the world's second leading cause of blindness globally and the leading cause of irreversible blindness: the World Health Organisation (WHO) estimated the incidence of blindness due to glaucoma to be 4.4 million people worldwide in 2002[11]. Unfortunately, the majority of glaucoma remains undiagnosed. Glaucoma is asymptomatic in early and moderate stages, and patients often present with manifestations of visual loss unless detected on examination. While elevated intraocular pressure (IOP) is no longer part of the definition of glaucoma, it is the sole proven modifiable risk factor for the development and progression of glaucomatous optic neuropathy[1]. Medical therapy is aimed at lowering IOP below a clinically determined target level in order to prevent or slow progression. However, in addition to the absolute IOP level, IOP fluctuations and, in particular, peaks, are related to progression[12]. These peaks tend to occur outside clinical office hours and frequently occur during the nocturnal sleep period. Hence, assessing IOP and, in particular, the IOP profile over 24 hours is pivotal in the management of glaucoma and its sequelae.

1.2 GLAUCOMA

Glaucoma is a heterogeneous group of progressive optic neuropathies, characterized by loss of retinal ganglion cells (RGC), leading ultimately to loss of vision and subsequent irreversible blindness. Early diagnosis is key, as the disease is treatable and its progression can be slowed or halted, while glaucomatous visual impairment is irreversible.

1.2.1 Pathophysiology

The aqueous humor is secreted by the non-pigmented epithelium of the ciliary body into the posterior chamber. It flows between the lens and the posterior iris, through the pupil and into the anterior chamber, before passing through the trabecular meshwork into Schlemm's canal and then into the aqueous veins of the episcleral venous system. Furthermore, a small amount of aqueous humor, independent of IOP, indirectly leaves the eye through the uveoscleral drainage.

The following mechanisms may play a role in the pathophysiology:

- Reduced availability of neurotrophic factors
- Localized vascular insufficiency in the retinal nerve head may lead to hypoxia of the optic nerve
- Pro-inflammatory cytokines produced by glial cells promote an innate immune response, potentially self-perpetuating, leading to degradation and adverse remodeling of the extracellular matrix[13]. It is thought that due to these induced

changes, the biomechanical effects impact on the structure of the optic nerve head[14].

- Optic disc cupping may further enhance the biomechanical effect on the optic nerve head[14].
- Neuroinflammation, a glial activity, is a phenomenon frequently observed in degenerative central nervous system disorders that is characterized by RGC death without mononuclear cell infiltration[15, 16]. Auto-antibodies against retinal antigens have been identified in patients with glaucoma, which suggest an innate rather than an adaptive immune response[17].

Clinically these structural changes, i.e. the topographical deepening and widening of the optic disc cup represent RGC axonal loss and adverse remodeling[14]. These glaucomatous changes are visible by ophthalmoscopy or with imaging technologies.

1.2.2 Classification and Definitions

According the European Glaucoma Society (EGS), open-angle glaucoma (OAG) is classified into primary or secondary forms based on the irido-corneal angle as assessed by gonioscopy, slit-lamp biomicroscopy, optic nerve head findings and the VF defects[18]. Primary open-angle glaucoma (POAG) is associated with:

- Evidence of optic nerve damage from either, or both, of the following
  - Optic disc or retinal nerve fibre layer (RNFL) structural abnormalities
  - Reliable and reproducible visual field (VF) abnormality
- Adult onset
- Open anterior chamber angles
- Absence of other known explanations (i.e. secondary glaucoma)

Although many patients with POAG present with elevated IOP as compared to the normal range (10-22 mmHg), nearly 40% of those with otherwise characteristic of POAG may not have elevated IOP measurements and are therefore called normal tension glaucoma (NTG) patients[19].

1.2.3 Epidemiology

Glaucoma is the world’s leading cause of irreversible blindness, the second leading cause of blindness globally. It is estimated that about 61 million people suffer from glaucoma worldwide[11], with 45 million people affected by POAG and 16 million by primary angle closure glaucoma (PACG). In 2020, the number of patient with PACG is expected to increase to 21 million, with 5.3 million bilaterally blind. The prevalence of POAG is 4.2% in black and 2.1% in white populations respectively[20]. The prevalence of glaucoma increases markedly with age. The significant increases of life expectancy in many populations worldwide means that the health burden from glaucoma is likely

to increase significantly in future years. This is for two reasons; firstly an older population will have a higher prevalence of glaucoma, secondly longer life expectancy means that individuals with glaucoma will have a longer ‘window of risk’, i.e. more time in which to develop visual loss.

In the Baltimore Eye Survey approximately half of all patients diagnosed with POAG had an initial IOP of less than 21 mmHg at the time of diagnosis. While participating in the survey, approximately 20% of patients had an IOP lower than 21 mmHg on each of the first three visits. Hence the prevalence of NTG is considerable[21, 22]. Glaucoma without elevated pressure may be even higher in some Asian countries[23]. There is no consensus on whether POAG and NTG are distinct types of OAG or pressure-defined subgroups of the same disease.

1.2.4 Treatment

The purpose of glaucoma treatment is to lower IOP below a clinically determined target level in order to prevent progression of the optic neuropathy and subsequent visual loss. Treatment is indicated for patients who are at risk for developing functional impairment or decrease in vision-related quality of life (QoL) from the disease, in particular when the risk of progressive disease outweighs the risks and potential side effects of treatment. Typically, based on the patient’s IOP, the glaucomatous changes of the optic disc, VF status and, if available, the rate of VF deterioration, a target IOP range is set, at which the physician estimates that development of further glaucomatous damage may be prevented or reduced to a minimum[18, 24]. The target IOP is the IOP range in which the ophthalmologist judges the estimated rate of progression is unlikely to affect the patient’s QoL. Once the IOP range has been determined, IOP-lowering treatment will be instituted to bring and maintain the IOP in that range. IOP can be reduced by medication, laser treatment or surgery.

1.2.4.1 IOP lowering by medication

Several classes of drugs are available to decrease IOP either by reducing aqueous humor production (beta-blockers, alpha-adrenergic agonists, carbonic anhydrase inhibitors such as timolol, brimonidine, acetazolamide) or by facilitating its outflow (prostaglandins, parasympathomimetics such as latanoprost, pilocarpine).The therapy usually starts as a monotherapy with the administration of a single drug. If the resulting IOP reduction is not sufficient, a combination of 2 or more drugs from different classes can be considered[25].

If medication does not prove effective, most patients will undergo laser treatment or surgery.

1.2.4.2 IOP lowering by laser treatment

The most common type of laser treatment performed for POAG is called trabeculoplasty. During Argon Laser Trabeculoplasty (ALT) laser spots are applied

circularly onto the trabecular meshwork, stimulating the opening of the meshwork to allow outflow of aqueous humor and lowering of IOP. Selective Laser Trabeculoplasty (SLT) utilizes a YAG laser to selectively target melanin within the pigmented trabecular meshwork cells, leaving untreated portions of the trabecular meshwork intact. Trabeculoplasty disadvantages include a low rate of patients responding to therapy, short duration of treatment effect and the persistence of IOP fluctuations despite a low mean IOP.

1.2.4.3 IOP lowering by surgery

Conventional surgery involves creating a drainage hole allowing the aqueous humor to bypass the clogged natural drainage canals and flow out of the eye through this new artificial drainage canal. Various more or less established forms of less invasive glaucoma surgery also exist.

1.3 SENSIMED GOLDFISH

GF is a pre-CE mark non-implantable medical device, consisting of a CL, an adhesive antenna and recording system, intended for continuous recording of IOP for up to 24h.

GF CL is molded from medical grade silicone elastomers with surfaces treated with oxygen plasma to ensure hydrophilicity. A Micro-Electro-Mechanical System (MEMS) pressure sensor an antenna and a telemetry microprocessor are embedded in the lens.

The CL is placed on the eye and sends its signals wirelessly to a recorder via a thin flexible data cable and a periorbital patched adhesive antenna. Upon completion, the recording is transmitted to a computer for read-out and visualization.

1.3.1 Initial testing with GF

GF CL underwent initial bench as well as internal testing described in the Investigator's Brochure

1.3.1.1 SENSIMED Goldfish Sensor (GF CL)

The silicone used for GF CL, and which will be in contact with the eye, is made by the same manufacturer and of the same product family as the one used for the SENSIMED Triggerfish Sensor.

1.3.1.2 SENSIMED Goldfish Antenna

The SENSIMED Goldfish Antenna is 100% equivalent to the approved and CE-marked SENSIMED Triggerfish® Antenna.

1.3.1.3 SENSIMED Goldfish Data Cable

The SENSIMED Goldfish Data Cable is 100% equivalent to the approved and CE-marked SENSIMED Triggerfish® Data Cable.

1.3.1.4 SENSIMED Goldfish Recorder

The SENSIMED Goldfish Recorder hardware is 100% equivalent to the approved and CE-marked SENSIMED Triggerfish® Recorder, with a firmware version that has been adapted to the new nature of the sensor.

1.3.1.5 SENSIMED Goldfish Software

The SENSIMED Goldfish Software is equivalent to the approved and CE-marked SENSIMED Triggerfish® Software, but has been adapted to visualize the different nature of the measured data.

1.3.1.6 Recorder Sleeve, Battery Charger and Bluetooth® USB

All additional materials used are 100% equivalent to the ones contained in the approved and CE-marked SESNIMED Triggerfish® system.

1.4 STUDY RATIONALE

The dynamics and repeatability of the 24-hour IOP indicates the existence of a nycthemeral pattern, following the day/night light cycle[1]. 24-hour IOP variations have been reported to be associated with VF progression loss[26]. These fluctuations have also been reported to be larger in glaucoma patients as compared to healthy subjects[2, 3, 24, 27].

The importance of the nycthemeral IOP pattern in the management of glaucoma has been well documented[28, 29], especially for patients who experience visual loss despite apparently normal and/or controlled IOP during office hours. Currently, the usual way of assessing nycthemeral IOP fluctuation is to perform repeated tonometry IOP measurements. These allow only snapshot and non-continuous measurements, once per hour in the best cases. Furthermore, these measurements are cumbersome, expensive, do not consider the IOP changes related to day-life activities and require awakening patients during the nocturnal/sleep period, thus disturbing the IOP nocturnal rhythm.

Automated and telemetric methods to continuously monitor IOP for 24 hours are therefore an important unmet need in glaucoma.

There have been many efforts in the past decades to search for an implantable permanent device or a removable temporary device to monitor IOP continuously in humans.

Many handheld, portable self-monitoring devices have been evaluated[4-6], however each of them seems to be inaccurate and technically challenging for older glaucoma patients[30]. Furthermore, none of them addresses the crucial issue of IOP behaviour during undisturbed sleep. Implantable telemetric devices have been developed for long-term

continuous IOP monitoring[7-9]. One such approach, developed by Downs et al., has demonstrated its ability to measure continuous IOP and ocular pulsation in non-human primates[7]. Todani et al. have also reported on the feasibility of a wireless transducer based on pressure-sensor cells for continuous IOP monitoring in rabbits[31]. Tested in human eyes, this technology showed promising results. It is however difficult to foresee the meaning or potential of this novel technique for improving the understanding of glaucoma[9]. Implantable devices requires surgical intervention. Moreover, important drawbacks of such IOP monitoring devices are the general inability of these devices to provide measurements during sleep period and the potential need for subsequent re-intervention in cases of device failure or malfunction.

A variety of non-implantable CL-based technologies are in development. This temporary IOP monitoring approach does not need surgical implantation, is easily reversible and offers the potential advantages to be used by many patients[32]. Greene and Gilman were the first to propose the use of a CL for IOP monitoring[33]. The major drawback was that the CL needed to be custom molded for each eye in order to detect small changes occurring at the meridional angle of the corneoscleral junction. In 2010, Twa et al proposed integrating the piezoresistive sensor tip of the dynamic contour tonometer (DCT) into a hard CL[10]. The sensor is located in a contoured probe, which is assumed to minimize the effect of corneal parameters on measurement errors. This approach provided reliable IOP measurements in a group of healthy volunteers for up to 100 seconds. Beyond the limited duration of measurements, there are other drawbacks of this approach, including the use of a hard CL, patient discomfort, the location of the sensor tip in the center of the CL with resulting decrease of vision, and the use of a wire.

In this context, Sensimed AG has developed a new device intended to measure IOP over 24 hours. GF includes a CL molded from medical grade silicone elastomers with surfaces treated with oxygen plasma to ensure hydrophilicity. A MEMS pressure sensor, an antenna and a telemetry microprocessor are embedded in the lens, allowing for continuous IOP recording. The CL is placed on the eye and sends its signals wirelessly to a recorder via a periorbital patched adhesive antenna. Upon completion, the recording is transmitted to a computer for read-out and visualization.

The purpose of this pilot study is to evaluate the safety and tolerability of the GF as well as its ability to detect IOP changes in healthy subjects and OAG patients.

1.5 GUIDANCE FOR INVESTIGATOR

The investigator is responsible for ensuring that no subject is exposed to any study-related examination or activity before giving written informed consent.

The investigator, or authorized co-investigator, will inform the subject of the aims, methods, anticipated benefits and potential hazards of the study, including any discomfort it may entail. These verbal explanations will cover all the points specified in the written information leaflet provided to the subject. The subject will be given every opportunity to request a clarification on any points he/she does not understand and, if necessary, to

ask for more information. At the end of the interview, the subject will be given time to reflect on his/her participation in the study. Subjects who accept participating in the study will be requested to sign and date the informed consent form (ICF). Subjects will receive copies of the written information leaflet and of the signed ICF.

The subject is at liberty to withdraw his/her consent to participate in the study at any time, without penalty or loss of benefits to which he/she is otherwise entitled. Subjects who refuse to give, or withdraw written informed consent may not be included or continued in the study.

Should new information become available during the investigation that may affect the subjects' decision to participate, this new information will be made available to the subjects in written and oral form by the investigator. When appropriate, subjects will be asked to sign an updated consent form if they wish to continue participating.

After completion of the study, ICFs as well as all the written information provided to study subjects, will be kept and archived in the Investigator Site File (ISF), according to the requirements of the country's health authorities, but for a minimum of 10 years after trial completion. The ICF will be translated to the customary language of the study center and adapted to the requirements of the local Ethics Committee (EC).

2 OBJECTIVES

The objective of this study is to investigate the use of GF for 24-hour IOP monitoring in healthy subjects and OAG patients

3 ENDPOINTS

3.1 PRIMARY ENDPOINT

- Changes in IOP assessed by GF following known physiological and induced changes in IOP

3.2 SECONDARY ENDPOINTS

- Percentage of subjects completing 24-hour session with GF
- Wearing discomfort of GF based on visual analogue scale (VAS)
- Evaluation of GF technical performance based on the percentage of valid GF measurements
- Correlation between IOP and ocular pulse amplitude (OPA) assessed by GF in the GF eye and IOP and OPA measured by tonometry in the fellow eye
- Relationship between GF IOP and blood pressure (BP) measurements over 24 hours

3.3 EXPLORATORY ENDPOINTS

- Comparison between healthy subjects and glaucoma patients with regard to IOP changes detected by GF
- Detection of body position changes using the accelerometers incorporated in the GF device

3.4 SAFETY ENDPOINT

AEs and SAEs will be collected throughout the duration of the study.

4 STUDY DESIGN

This is a prospective pilot study to investigate the safety and tolerability of the GF as well as its ability to detect physiological and induced IOP changes in healthy subjects and OAG patients.

Glaucoma subjects will be selected based on scheduled appointments (consecutive selection) and invited to participate in the study or could be recruited through advertising. The healthy subjects could also be recruited through advertising or among persons accompanying patients to clinic appointments.

After having signed and dated the subject informed consent form, subjects will undergo an initial ophthalmic examination. Subjects presenting average ocular parameter values such as a central corneal radius (CCR; flat meridian) between 7.5 mm (45 D) and 7.9 mm (42.75 D) and a central corneal thickness (CCT) between 500 µm and 600 µm in both eyes will be eligible for the study. Subject will also need to present a good IOP symmetry between eyes, as defined in the inclusion criteria to be qualified for the study. The GF eye will be randomly selected and tested for GF CL adhesion.

In line with international and American national standard for ophthalmic instruments, to demonstrate the range of IOP values measurable with GF, subjects will be recruited to be evenly allocated to one of the 3 different groups based on their mean IOP value at screening acquired with GAT:

- IOP between 7 and 16 mmHg (group 1)
- IOP between 17 and 22 mmHg (group 2)
- IOP equal or higher than 23 mmHg (group 3).

Glaucoma subjects will be either naïve to IOP-lowering drug treatment or washed out thereof for a 4-week period before the recording session. In case of washed-out patients, IOP will be reassessed before allocation to one of the IOP groups. If allocation is not possible, patient will be withdraw and replaced.

After baseline ophthalmic examination, the VAS will be assessed. GF CL will then be placed on the GF eye for a 24-hour recording session, simultaneous to a 24h BP recording.

During the session, each subject will undergo provocative tests (PT) including postural changes and water drinking test (WDT) that are known to induce a change in IOP. IOP will be measured in the fellow eye at specific time points as well as during the PT, using a tonometer. This will allow comparison between fellow eyes as IOP by tonometry is not assessable in the GF eye during recording. As much as possible, the same operator will measure the IOP for a specific subject. Following the PT, the recordings will be continued in ambulatory conditions with the subjects staying calm at home in order to minimize activity-related variation of IOP. Subject activities will be captured in a logbook. Subjects will also be asked to refrain from lying down except during the nocturnal sleep period to avoid uncontrolled variation of IOP. Upon completion of the session, wearing discomfort will be scored on a VAS. GF and BP monitor will be removed and a final ophthalmic examination will be conducted to exclude or adequately address any adverse events and return the logbook to the investigator. This concludes the study for the subjects.

The overall study duration for an eligible subject is limited to 2 days, if no washed out is needed, 4 weeks otherwise.

A sample size of 12 subjects was estimated to evaluate the correlation between GF and GAT with a significant statistical power. Therefore, the study has been planned to recruit at least 12 eligible subjects, 6 healthy subjects (4 in group 1 and 2 in group 2) and 6 patients with glaucoma (2 in group 2 and 4 in group 3).

It is assumed that the recruitment of 12 eligible subjects is completed within 5 weeks from the date of initiation.

Hence, the overall study duration from the first subject accrued until last subject out of the study equates to about 6 weeks. A longer recruitment period may be allotted to allow for sufficient subject enrolment.

5 STUDY POPULATION

To fit the criteria for analysis each subject must at least:

- Meet all of the inclusion and not meet any of the exclusion criteria specified in the following sections within the specified time frame for the baseline visit, or benefit from a Sponsor-accepted waiver from one or more inclusion and/or exclusion criteria (if applicable)
- Complete the required activities specified in the protocol (see section 6), and
- Have his/her Case Report Form (CRF) completed, received and accepted by the Sponsor

5.1 SUBJECTS

The study POAG population reflects the target population for the device, while the healthy population constitutes a non-glaucomatous reference group.

5.2 ELIGIBILITY CRITERIA

To be eligible for inclusion into this study, each subject must fulfil all of the following criteria at latest on study day 0 (SD0) before being enrolled in the study.

5.2.1 Inclusion Criteria (for both eyes where applicable):

Subjects are eligible for inclusion if all the following criteria are fulfilled for the GF eye, when applicable:

1. For OAG subjects,
    a. a clinical diagnosis of POAG, including NTG with glaucomatous structural defects, glaucomatous VFs, and open angles on gonioscopy
    b. all known untreated IOP measurements < 22 millimeters of mmHg using GAT for NTG
    c. no IOP-lowering treatment; otherwise, a 4-week wash-out period prior to the recording
2. For healthy subjects, no structural defects, normal VFs, IOP ≤ 21 mmHg and open angles on gonioscopy
3. Aged ≥ 18 years, either gender
4. BMI ≤ 30 kg/m2
5. CCR (flat meridian) between 7.5 mm (45 D) and 7.9 mm (42.75 D)
6. CCT between 500 μm and 600 μm
7. Difference in IOP absolute value between eyes within 2.5 mmHg at screening in sitting position
8. Same direction of IOP variation (positive or negative) for the 2 eyes when moving from sitting to supine positions at screening
9. Spherical refraction within ±6.00 diopters and cylinder refraction within ±3.00 diopters
10. Having given written informed consent, prior to any investigational procedures

5.2.2 Exclusion Criteria

Subjects are to be excluded from the study if any of the following criteria is fulfilled:

1. Ocular pathology (other than glaucoma for glaucoma subjects)
2. Previous glaucoma, cataract or refractive surgery
3. Corneal or conjunctival abnormality, precluding contact lens adaptation
4. Severe dry eye syndrome

5. Subjects with allergy to corneal anesthetic
6. Subjects with contraindications for silicone CL wear
7. Subjects with contraindications for WDT (eg., heart, renal problems)
8. Subjects unable or unwilling to comply with the study procedures
9. Participation in other interventional clinical research within the last 4 weeks

A waiver to specific exclusion criteria may be obtained through the study's Medical Responsible. The Investigator completes a waiver request form (WRF) that upon agreement and signature by the Medical Responsible will be kept in the Trial Master File (TMF).

5.3 ASSIGNMENT TO STUDY GROUPS

12 eligible POAG patients and 12 healthy subjects will be enrolled. Informed consent will be obtained from all subjects prior to the performance of any study related procedures.

Subjects are to be identified by their initials before a subject number is allocated.

When subject has been confirmed to be eligible for the study a unique non-identifying study identification number will be allocated by the sponsor in sequential, chronological order and by study arm.

6 STUDY PROCEDURES AND ASSESSMENTS

All assessments apart from GF will be performed using the investigational site's equipment. All equipment should be appropriately calibrated and maintained according to manufacturers' instructions throughout the study and for all study assessments.

All procedures and assessments will be performed according to the study schedule (see Appendix A).

6.1 OUTLINE OF STUDY PROCEDURES AND ASSESSMENTS

6.1.1 Screening

- Demographic data
- Medical and ophthalmic history data
- Ophthalmic examination (both eyes)
  - VF test (Humphrey 24-2 SITA Standard)
  - RNFL thickness by OCT
  - Corneal epithelial thickness by OCT

    - Gonioscopy
    - Slit lamp examination
    - Refraction test
    - Axial length
    - Topography/keratometry
    - Pachymetry
    - IOP by pneumatonometer in sitting and supine positions
    - Corneal biomechanics by ORA
  - Height
  - Weight
  - Reassessment of IOP for washed-out OAG patients
  - Check of all eligibility criteria
  - GF eye selection (random)
  - GF CL adhesion testing on the GF eye

6.1.2 SD0

Study procedures will start between 10:30 am and 12 am. GF and BP recording will be initiated simultaneously

The following procedures will be carried out:

  - Ophthalmic examination before GF-CL placing (both eyes)
    - Slit lamp examination (if SD0 different from screening)
    - IOP by GAT in sitting position
    - IOP by DCT in sitting position
    - IOP by Pneumatonometer in sitting position
  - VAS assessment
  - Off-eye measurement of the GF CL
- GF-CL placing in the GF eye (between 11 am and 12:30 am) and ensure correct fitting
  - BP monitor placing (between 11 am and 12:30 am)
  - Following procedures as below (full 24h monitoring):

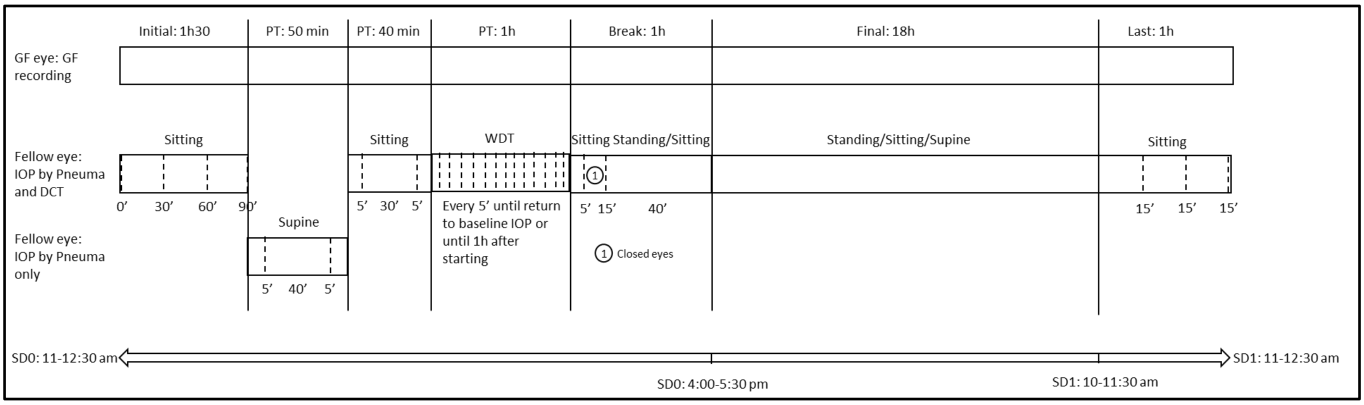

- WDT
  - Subject will be asked to drink 1.0 L of water within 5 min
  - IOP in sitting position every 5 min after starting the WDT until return to baseline IOP (± 1 mm Hg) or until 1h after starting the WDT, whichever comes first
- GF recording in the GF eye under ambulatory conditions until 24 hours after the start of the recording
  - Patient booklet
  - Subject logbook
- Concomitant medication and procedures
- AEs and device deficiencies

The recording will be carried out in ambulatory conditions with the study subjects staying calm at home. Subject activities will be captured in a patient logbook.

Full-metal frame glasses (vision correction, reading, sunglasses…) are prohibited throughout the recording since they block the data transfer from the Sensor to the recorder.

If the subject wishes to read during the recording session, the reading material should be placed at eye level (Figure 1) to assure communication between the lens and the antenna (see device description).

Figure 1 Recommendations for reading activity during TF recording

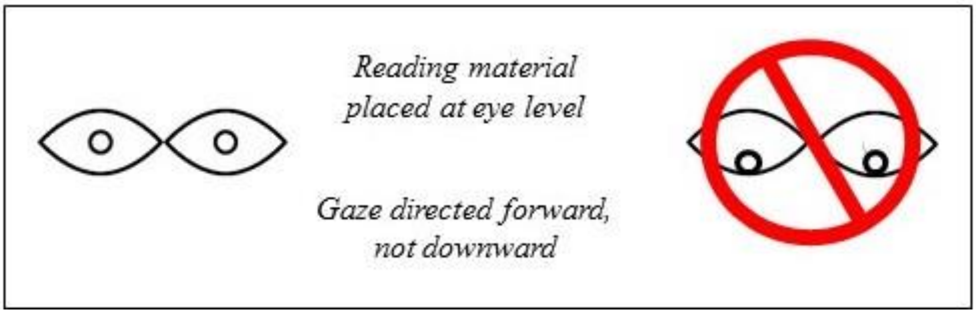

6.1.3 SD1

Subjects will be requested to arrive at the hospital 1h before the end of the recording session.

- GF recording in the GF eye will be continued:
    - Subject logbook
    - IOP measurements in the fellow eye as described in the procedure above
- BP recording
- VAS assessment
- GF-CL removal with tweezers 15 min before the end of the recording session
- Off-eye measurement of the lens right before the end of the recording session
- BP monitor removal at the end of the session
- Ophthalmic examination (both eyes)
    - RNFL thickness by OCT
    - Corneal epithelial thickness by OCT
    - Slit lamp examination
    - Axial length
    - Topography/keratometry
    - Pachymetry
    - IOP by GAT in sitting position
    - IOP by DCT in sitting position
    - Corneal biomechanics by ORA
- Concomitant medication and procedures

- AEs and device deficiencies since last visit
- Download of recorded data to the investigator's computer

6.2 SUBJECT COMPLETION AND WITHDRAWAL

Subjects will be informed that they have the right to withdraw from the study at any time, without prejudice to their medical care, and that they are not obliged to state their reasons.

Any withdrawal must be fully documented in the CRF and source documents, and should be followed up by the Investigator.

The Investigator may withdraw a subject at any time if this is considered to be in the subjects' best interest.

Withdrawal is mandatory in the following situations:

- Any SAE thought to be attributable to GF (subject will be followed until event resolves or stabilizes or until 30 days after the subject's last exposure to the investigational device, whichever comes first)
- Subject is unable or unwilling to comply with the protocol
- Requests by the subject to be withdrawn from the study
- Requests by the investigator to withdraw the subject from the study
- Severe protocol violations
- No adaptable GF CL on the subject's selected eye
- Subject cannot be allocated to any of the group

Subjects will be considered as having completed the study according to protocol.

If study participation is prematurely discontinued, the primary reason for discontinuation must be recorded in the appropriate section of the CRF. All efforts will be made to complete and report the observations following study discontinuation as thoroughly as possible.

6.3 REPLACEMENT POLICY

The following eligible subjects will be replaced:

- A subject who prematurely withdraws or is discontinued from the study before having a GF recording.
- A subject who has been withdrawn from the study because no GF CL could be placed on his/her eye
- A washed-out OAG patient who cannot be allocated to any of the group

6.4 CONCOMITANT MEDICATIONS AND THERAPIES

All concomitant medications and therapies taken, changed or administered during the study will be documented in the subject clinical/hospital and study records.

No IOP-lowering treatment is permitted during the course of the study. For OAG patients who are on an IOP-lowering treatment regime at screening, a 4-week wash-out period is mandatory prior to the recording session. Subjects will be given standard ophthalmologic anaesthetic drops and artificial tear drops customary at the investigational site.

6.5 OBSERVATION AND MEASUREMENTS

The following measurements and observations will be made to assess the various endpoints:

- 24-hour IOP recording with GF
- VAS
- 24-hour BP recording
- AEs and SAEs

7 SENSIMED GOLDFISH

7.1 DEVICE DESCRIPTION

GF is a sensing CL-based device intended to monitor IOP continuously over a period of 24 hours, to aid in the management of glaucoma. It is non-invasive and intended to be used during wake and sleep periods.

The GF Set consists of:

1. SENSIMED Goldfish Sensor
2. SENSIMED Goldfish Antenna
3. SENSIMED Goldfish Recorder
4. SENSIMED Goldfish Data Cable
5. SENSIMED Goldfish Software
6. Recorder Sleeve
7. Battery Charger
8. Bluetooth USB (Universal Serial Bus) Stick

To minimize differences in human performance, the device is to be operated only by qualified health care professionals who have been trained by Sensimed representatives in the handling and operation of GF.

Please consult the information concerning the device in the “SENSIMED Goldfish Investigator’s Brochure” that is provided as a separate document.

7.2 MANUFACTURER

GF is manufactured by:

Sensimed AG
Route de Chavannes 37
1007 Lausanne, Switzerland

GF-CL, Recorders and Data Cables are assigned individual ID numbers in addition to lot numbers. Antennae and Sleeves carry a lot number and Software carries a revision number.

All devices provided for the study, including disposable and re-usable items, are Sponsor property. All unused disposables and re-usable items are to be returned to Sponsor at the end of the study.

8 SAFETY

8.1 SUMMARY OF OVERALL RISK AND BENEFITS

8.1.1 Anticipated Risks

The risks correspond to the GF CL wear on the eye for up to 24 hours. The lens material is similar to that of commercially available soft silicone contact lenses.

The risk consists of AE due to CL wear including pain, injection, temporary reduction of BCVA, ortho-K effect, discomfort, conjunctival and corneal structural alterations or infections, dry eye sensation and corneal swelling.

A complete eye examination will be done prior to GF CL placing to exclude risk factors that could place the subject at increased risk for ocular complications, and immediately after CL removal for appropriate detection and treatment of any device-related side effects. Subjects in this study will be provided a direct medical contact that can be reached throughout the recording session with GF.

The adhesive patch containing the periorbital antenna may cause local skin irritation or, if used on very sensitive skin, local inflammation. Known or unknown allergy to any material in the antenna can provoke an allergic response.

Risks related to other ophthalmologic examinations are referred to the Investigator.

8.1.2 Anticipated Clinical Benefits

There will be no immediate benefit to the subjects participating in this study; however, the risks to study subjects in the proposed study have been minimized to the extent possible, and are reasonable in relation to the importance of the knowledge that may be obtained by this study.

8.2 DEFINITION OF AN ADVERSE EVENT

An AE is defined as any untoward medical occurrence in a subject. AEs are categorized according its causal relationship to GF exposure (i.e. not related or related) and whether it is a SAE (i.e. serious or not).

Categories of adverse events			
**adverse events**	**non-device related**	**device or procedure related**	
non-serious	adverse event (AE)	adverse device event (ADE)	
serious	serious adverse event (SAE)	serious adverse device event (SADE)	
		anticipated	unanticipated
		ASADE	USADE

8.3 DEFINITION OF A SAE

The definition of a SAE is any untoward medical occurrence that:

- Led to death;
- Led to a serious deterioration in the health of the patient
  - Resulted in life-threatening illness or injury
  - Required in-patient hospitalization or prolongation of existing hospitalization;
  - Resulted in permanent impairment of a body structure or a body function
  - Resulted in medical or surgical intervention to prevent permanent impairment of a body structure or a body function
- Led to fetal distress, fetal death, or a congenital abnormality or birth defect

SAEs are categorized according its causal relationship to GF (i.e. not related or related). A device-related SAE, a serious adverse device effect (SADE) is further categorized as anticipated (ASADE) or unanticipated (USADE). A USADE is any SAE that has an effect on health or safety or any life-threatening problem or death caused by, or associated with, a device, if that effect, problem, or death was not previously identified in nature, severity, or degree of incidence in the investigational plan or application (including a supplementary plan or application), or any other unanticipated serious problem associated with a device that relates to the rights, safety, or welfare of subjects.

8.4 ABNORMAL TEST FINDINGS

Abnormal baseline values and conditions will not be considered as AEs. Any change in test findings and pre-existing conditions during the investigation will be considered AEs and reported as such, provided that they are judged clinically significant by the investigator and unless an expected evolution of the condition in question.

8.5 DEVICE DEFFICIENCIES

A device deficiency is defined as inadequacy related to identity, quality, durability, reliability, safety or performance of GF. These include malfunctions, use errors, inadequate labelling and deficiencies that might have led to a medical occurrence if:

- Suitable action had not been taken
- Intervention had not been made
- Circumstances had been less fortunate

8.6 SEVERITY GRADING FOR ADVERSE EVENTS

The investigator will assess all AEs. The severity should be graded into one of five classes describing the clinical severity of the event as it occurred. The grade of five classes of severity are:

- Mild AE (grade 1): symptom(s) barely noticeable to the patient or does not make the patient uncomfortable. The AE does not influence performance or functioning. Prescription drugs are not ordinarily needed for relief of symptom(s).
- Moderate AE (grade 2): Symptom(s) of a sufficient severity to make the patient uncomfortable. Performance of daily activities is influenced. Treatment of symptom(s) may be needed.
- Severe AE (grade 3): symptom(s) of a sufficient severity to cause the patient severe discomfort. Severity may cause cessation of treatment with the drug. Treatment for symptom(s) may be given
- Immediately life-threatening AE (grade 4)
- Fatal AE (grade 5)

8.7 RELATIONSHIP OF AES TO GF

The relationship of an AE to the GF device is assessed and determined by the investigator or designee after careful consideration of the event in terms of biological plausibility, possible extraneous causes, any pre-existing medical conditions or concomitant medications, temporal relationship between exposure to investigational product and the onset (or worsening) of the event, and known patterns of response to the GF device in general.

Assessment of the relationship is based on the following guidelines:

- Unrelated AE: the AE has no temporal (timing) relationship to the intervention and is not related to GF
- Unlikely related AE: the AE has no temporal relationship to the intervention and is unlikely related to GF
- Possibly related AE: the AE has a reasonable temporal relationship to the intervention and may be related to GF
- Probably related AE: the AE has a reasonable temporal relationship to the intervention and is likely related to GF
- Definitely related AE: the AE has a reasonable temporal relationship to the intervention and is clearly related to GF

8.8 COLLECTING, RECORDING AND REPORTING OF AES AND DEVICE DEFICIENCIES

Study subject will be routinely questioned about AEs at study visits. All observed or volunteered device deficiency, AEs (SAE or not) subject symptoms problems and complaints as well as abnormal test findings, regardless of treatment group, if applicable, or suspected causal relationship to GF or, if applicable, other study treatment or diagnostic product(s) will be recorded in the subjects' CRF using standard medical terminology in order to avoid the use of vague, ambiguous, or colloquial expressions.

For all AEs, sufficient information will be pursued and/or obtained so as to permit

- Adequate determination of the outcome of the effect (i.e. whether the event should be classified as a SAE)
- Assessment of the causal relationship between the AE and the GF or, if applicable, the other study treatment or diagnostic product(s).

AEs felt to be associated with the GF will be followed until the event (or its sequelae) or the abnormal test finding resolves or stabilizes at a level acceptable to the investigator or until 30 days after the subject's last exposure to the investigational device, whichever comes first.

SAEs, whether SAE, ASADE or USADE must be reported immediately without undue delay within 24 hrs to Sensimed AG:

Phone: +41 21 621 9191

Fax: +41 21 621 9193

Email: safety@sensimed.ch

The appropriate CRF pages should be updated with any information collected while reviewing the SAE in a timely manner. The Medical Responsible of the study (see protocol cover page) is available to provide medical guidance regarding SAE reporting.

ASADEs and USADEs are to be reported by the investigator, to the competent Institutional Review Board (IRB)/Ethics Committee (EC), within 10 working days of gaining knowledge of the event if there is a possible, probable or definite causal relationship with the investigational device.

For purposes of this study, Sensimed or its delegated agent will assume the responsibility of reporting AEs to the appropriate regulatory agencies.

AEs and device deficiencies are collected and recorded on an ongoing basis from baseline (pre-study screening) visit until the completion of the study.

New protocol related device deficiencies and AEs (caused by any intervention required by the protocol) and updates on AEs with an ongoing or unknown outcome must be recorded until the last subject visit required by the protocol. Beyond this reporting period, any new unsolicited serious AE spontaneously reported to the sponsor by the Investigator would however be collected and processed.

Within the study, all subjects who were exposed to the GF - whether they completed the recording session or not - should enter the AE and device deficiencies recording period as defined above.

All ongoing/unknown outcome AEs and device deficiencies will be followed-up until the last study visit. If a subject is documented as lost-to follow-up, ongoing/unknown outcome AEs or device deficiencies will not be followed-up. A last batch of queries will be sent after last study visit if remaining ongoing/unknown outcomes of reported AEs or device deficiencies are pending.

After the last batch of queries with all collected data has been fully processed, CRFs and the database will no longer be updated. Only SAEs and medically relevant ongoing/unknown outcome AEs will be followed-up until resolution or stabilization or until 30 days after the subject was last exposed to TF, whichever comes first.

Abnormal baseline values as well as intercurrent illnesses, including signs/symptoms of a pre-existing disease state will not be considered AEs. Only worsening of baseline conditions will be considered, and hence reported, as AEs.

9 STATISTICAL CONSIDERATIONS

Data management and statistical analysis will performed by Sensimed AG. The final analysis will be performed after the database has been locked.

9.1 OVERVIEW

This is a pilot study to evaluate the safety and tolerability of the GF as well as its ability to detect IOP changes in healthy subjects and OAG patients.

All subjects will be undergoing the same procedures. The primary outcome is the evaluation of the ability of the GF to record known physiological and induced variation in IOP over

24 hours. IOP variation will be assessed and compared to standard tonometry measurements acquired in the fellow eye, using statistical tests.

9.2 DESIGN CONSIDERATIONS

This is the first formal study assessing the use of GF for 24-hour IOP monitoring. IOP measured by tonometry in the GF eye right before GF placement and right after GF removal will be compared to IOP measurements acquired by GF at lens placement and removal. Furthermore, GF measurements acquired in the GF eye will be compared to corresponding tonometry measurements at each time point in the fellow eye.

9.3 ANALYSIS POPULATIONS

9.3.1 Safety Analysis Populations

The safety analysis population will consist of all enrolled subjects.

9.3.2 Performance Analysis Population

The performance analysis population will include subjects showing IOP changes of at least 3 mmHg (based on tonometry measurement) following at least 1 provocative test. This will ensure that the change in IOP is effective. To be considered reliable GF measurements should be available for at least 80% of the recording.

Missing data will not be imputed; i.e. only observed data will be used.

9.4 SAMPLE SIZE CONSIDERATIONS

Sample size for this pilot study is based on an equivalence study design with the hypothesis being that the IOP measurements with GF are within 5 mm Hg of the GAT/PT measurements at greater than 80% of the time points over which the measurements are performed. With IOP measurements being planned at 25 time points over the 24-hour period, 80% of this would be 20 time points. The alpha error for the sample size calculation was therefore adjusted for 20 comparisons using the Bonferroni correction and was fixed at 0.25% instead of the traditional 5%. Under the assumption that the standard deviation for GF measurements is equal to 2.5 mmHg, a sample size of 10 eligible subjects would give a power of 80% to detect an equivalence margin of 5 mm Hg between GF and GAT. Considering a dropout rate (non-availability of measurements over the 80% of the recording or intolerance to the sensor) of 10-20%, the final sample size considered was 12 subjects.

9.5 ENDPOINTS

9.5.1 Safety endpoint

AEs and SAEs will be collected on all subjects throughout the duration of the study.

9.5.2 Performance endpoints

9.5.2.1 Primary endpoints

- Changes in IOP assessed by GF following known physiological and induced changes in IOP

9.5.2.2 Secondary endpoints

- Percentage of subjects completing 24-hour sessions using GF
- Wearing discomfort of GF based on VAS
- Evaluation of GF technical performance based on the percentage of valid GF measurements
- Correlation between IOP and OPA assessed by GF in the GF eye and IOP and OPA measured by tonometry in the fellow eye
- Relationship between GF IOP and BP measurements over 24 hours

9.5.2.3 Exploratory endpoints

- Comparison between healthy subjects and glaucoma patients with regard to IOP changes detected by GF
- Detection of body position changes using the accelerometers incorporated in the GF device

9.6 STATISTICAL ANALYSIS

9.6.1 Overview

Analyses will be descriptive in nature. Results will be presented by health status (healthy, glaucoma) when applicable. IOP measured by tonometry in the GF eye right before GF placement and right after GF removal will be compared IOP measurements acquired by GF at lens placement and removal. Also, GF measurements acquired in the GF eye will be compared to corresponding tonometry measurements at time point in the fellow eye using statistical tests. Initial analysis will be performed using Student t test and the interpretations would be performed after Bonferroni correction.

All statistical analyses will be performed using Stata version 13.1 (StataCorp, College Station, Tx) statistical software. Other software such as R or Python might also be used.

9.6.2 Safety

Safety events will be summarized as proportions with associated 95% confidence intervals.

9.6.3 Performance

All statistical analysis will be carried out using two-sided tests. 95% confidence intervals will be provided for point estimates of performance.

The primary hypothesis for this study will be to demonstrate that the changes in IOP assessed by GF following known physiological and induced changes in IOP have equivalent values ± 5 mmHg than measurements acquired with standard tonometer for values acquired at before and after the GF lens placement in the GF eye and for at least 80% of the time points in the fellow eyes.

H_0: IOP difference between GF in the GF eye and GAT in the fellow eye is > +5 mm Hg or < -5 mm Hg for both starting and end of the recording session AND

IOP difference between GF in the GF eye and GAT in the fellow eye is > +5 mm Hg or < -5 mm Hg for at least 80% of the time points

Ha: IOP difference between GF in the GF eye and GAT in the fellow eye is within ± 5 mmHg for both starting and end of the recording session AND

IOP difference between GF in the GF eye and GAT in the fellow eye is within ± 5 mmHg for at least 80% of the time points

Student’s t test will be used to test and reject the null hypothesis with appropriate correction for the alpha error.

Detailed analyses including secondary and exploratory analyses will be specified in the statistical analysis plan.

10 STUDY ADMINISTRATION

10.1 REGULATORY AND ETHICAL CONSIDERATIONS

This study is to be performed in accordance with the protocol, the Declaration of Helsinki, International standards organization (ISO) 14155:2011 “Clinical investigation of medical devices for human subjects – Good Clinical Practice”, and all locally applicable regulatory requirements.

It is however industry and Sensimed standard to follow International Conference of Harmonisation Harmonized Tripartite Guideline for Good Clinical Practice (ICH/GCP). In accordance, before a subject can be screened or enrolled in the study, he/she must give written informed consent in accordance with aforementioned guidance documents.

10.1.1 Regulatory Authority Approval

Before initiation of the study at a center, written approval of the protocol, ICF and any information presented to potential study subjects must be approved by the appropriate IRB/EC. If any amendments to any of these documents occur during the study, written approval must be obtained prior to their implementation. The investigator is responsible

for ensuring that these actions occur. Where required by local regulations, the sponsor is responsible for ensuring IRB/EC approval of the study.

10.1.2 End of the Study

For administrative and safety reporting purposes the end of the study will be defined as the date of the final clinical database lock. This provides for a single and conservative definition.

10.2 INVESTIGATOR RESPONSIBILITIES

The investigator, a qualified expert, must be familiar with and conduct the study according to the protocol, ISO 14155:2011, ICH/GCP guidelines as well as local regulatory and IRB/EC requirements, as applicable.

The investigator is not allowed to deviate from the protocol, unless the deviation has as purpose to protect subject rights, safety and well-being. All protocol deviations will be recorded on the appropriate form.

This is a company-sponsored investigation. The investigator will be responsible for maintaining and updating the ISF.

10.2.1 Principal Investigator

The principal investigator will:

- Act as a representative for sub-investigators for decisions and discussions regarding this study as required
- As a qualified expert to assure appropriate medical input and advice relating to study design and execution
- Be responsible for the primary review and sign-off of the final report on behalf of all investigators
- First author on primary publication

10.2.2 Subject Confidentiality

The investigator must ensure that the subjects’ anonymity is maintained. On the CRFs or other documents submitted to the Sponsor or its designee, the subjects must only be identified by an assigned identification number and initials, and never be identified by their name. If subject names are included on copies of documents submitted to the Sponsor or the Sponsor’s designee, the names (except for initials) must be obliterated and the assigned subject numbers added to the documents.

The investigator should keep a separate log of subjects’ identification numbers, names, addresses, telephone numbers and hospital numbers (if applicable). Documents that are

not intended for submission to the Sponsor, e.g. the subject identification log and the signed ICF, must be maintained by the investigator in strict confidence.

10.3 DATA MANAGEMENT

The investigator or designee will be responsible for recording study data in the CRF provided by the sponsor. It is the investigator's responsibility to ensure the accuracy of the data entered in the CRFs.

CRFs will be completed for eligible subjects and enrolled subjects only.

The data will be entered into a database. Sensimed AG will be responsible for data processing.

10.4 STUDY MONITORING

10.4.1 Monitoring Visits

Sensimed AG will conduct a study initiation visit before enrolment of the 1st subject. During this visit the site investigational team will be trained on all study-related procedures.

Sensimed AG will conduct periodic monitoring visits during the course of the study reviewing the CRFs and other study documents and conducting source data verification, in order to verify that the study is conducted in accordance with the study protocol, ISO 14155:2011, ICH/GCP (as appropriate) as well as regulatory requirements and that the data are authentic, accurate and complete. The investigator must ensure that CRFs are completed in a timely manner and must allow the Sponsor access to subject records and all study-related materials during these visits. The extent of and frequency of monitoring visits will be determined by factors such as the design and complexity of the study and the site enrolment rate and data quality, using a risk-based approach, if appropriate, and prospectively identifying elements that are critical to study data quality and integrity.

Upon study completion, the Sponsor will visit the site to conduct a study termination visit. This will involve collection of any outstanding documentation.

Detailed procedures associated with study monitoring will be documented in a separate Monitoring Plan.

10.4.2 Protocol Deviation

All detected and recorded protocol deviations should be classified as major or minor. Generally, major deviations are those that affect the safety of subjects, or the scientific integrity of the study and its outcome.

Examples of major protocol deviations include:

- Failure to obtain informed consent

- Informed consent obtained after the initiation of study procedures
- Use of an unapproved ICF
- Enrolling subjects who are not eligible according to the IRB/EC approved protocol and for whom the sponsor has not provided a waiver
- Omitting study procedure(s) required by approved protocol and related to key study endpoints
- Failure to report an SAE
- Failure to follow the Safety Monitoring Plan

A minor protocol deviation is a protocol violation that does not adversely affect the risk/benefit ratio of the study, the rights, safety, or welfare of the subjects or others, or the integrity of the study. Examples of minor protocol deviations include:

- Study procedure conducted out of timeframe
- Copy of consent form not given to subject during informed consent process
- Subject failure to return logbook
- Missing original signed consent, but have a copy of the subject signed consent

10.5 QUALITY ASSURANCE

In compliance with ISO 14155:2011, ICH/GCP and regulatory requirements, the Sponsor, a third party on behalf of the Sponsor, regulatory agencies or IRB/ECs may conduct quality assurance audits at any time during or following a study. The investigator must agree to allow auditors direct access to all study-related documents including source documents, and must agree to allocate his or her time and the time of his or her study staff to the auditors in order to discuss findings and issues.

10.6 STUDY OR SITE DISCONTINUATION

The Sponsor may temporarily or permanently discontinue the study at the site for safety, ethical, compliance or other reasons. If this is necessary, the Sponsor will endeavour to provide advance notification to the site. If the site or study is suspended or discontinued, the investigator will be responsible for promptly informing the IRB/EC. Where required by local regulations, the Sponsor will be responsible for informing the IRB/EC of study or site discontinuation. In such cases, all study data and unused and re-usable GF items must be returned to the Sponsor.

10.7 RETENTION OF ESSENTIAL STUDY DOCUMENTS

Essential documents as defined by ISO 14155:2011 and ICH/GCP i.e. the signed protocol and any amendment(s), copies of the completed CRFs, signed ICFs from all subjects who consented, hospital records, logbook and other source documents, IRB/EC approvals and

all related correspondence including approved documents, device accountability records, study correspondence and a list of the subjects' names and addresses, will be maintained in the TMF/ISF and updated as appropriate throughout the study.

The investigator must retain copies of the essential documents for at least 10 years as specified by Sensimed and longer if required by applicable regulatory requirements (in accordance with ISO 14155:2011 and ICH/GCP).

The investigator will inform the Sponsor of the storage location of the essential documents, and must contact the Sponsor for approval before disposing of any. The investigator should take measures to prevent accidental or premature destruction of these documents.

10.8 AGREEMENTS AND INSURANCE

A clinical trial agreement between the Sponsor and investigator will be used as a separate study related document. Where required, Sensimed provides a liability insurance certificate as a separate study related document.

11 PUBLICATION

There will be a single analysis of the data as this is a single centre study. Hence the principal investigator will be offered to be the designated first author, whereas sub-investigators and relevant staff will be assigned authors, as determined by the principal investigator. In addition, contributing personnel of Sensimed may in consensus with the principal investigator be included in the authorship. The final manuscript will be prepared in conjunction with Sensimed.

Any manuscript, abstract or other publication or presentation of results or information arising from the study (including ancillary studies involving trial subjects) must be prepared in conjunction with Sensimed. Such materials must be submitted to Sensimed for review and comment at least 30 days prior to submission for publication or presentation.

12 STUDY TIMETABLE

- Projected starting date : May 2018
- Projected number of eligible subjects : 12
- Projected completion of subject accrual : June 2018
- Projected study end date : July 2018

13 APPENDIX A

STUDY SCHEDULE

Procedures	Screening	SD0	SD1
Informed consent	X		
Demographics	X		
Medical and ophthalmic history	X		
VF test (24-2 SITA Standard)	X		
OCT-RNFL and corneal epithelial thickness	X		X
Gonioscopy	X		
Slit lamp examination	X	X	X
Refraction	X		
Axial length	X		X
Topography/Keratometry	X		X
Pachymetry	X		X
IOP by Pneumotonometer	X[1]	X[2,3]	
ORA	X		
Height and Weight	X		
Inclusion and exclusion criteria	X		
GF eye selection	X		
GF CL adhesion test	X		
IOP by GAT		X[2]	X[2]
IOP by DCT		X[2]	X[2]
Off-eye lens measurement		X[4]	X[5]
24-hour GF recording		X	X[6]
24-hour BP recording		X	X
PT		X	
Patient booklet		X	
Subject logbook		X	X
VAS			X
GF and BP data download			X
Concomitant medication	Throughout the study		
AE	Throughout the study		
Device deficiencies		X	X

[1]Sitting and supine positions, both eyes
[2]Sitting position, both eyes
[3]Supine position, fellow eye
[4]Before placing the GF CL on the GF eye
[5]Before the end of the recording session
[6]GF CL removal 15 min before the end of the recording session

14 REFERENCES

1. Weinreb RN, Khaw PT. Primary open-angle glaucoma. Lancet 2004;363(9422):1711-20.

2. Liu JH, Kripke DF, Twa MD, et al. Twenty-four-hour pattern of intraocular pressure in the aging population. Invest Ophthalmol Vis Sci 1999;40(12):2912-7.

3. Liu JH, Zhang X, Kripke DF, Weinreb RN. Twenty-four-hour intraocular pressure pattern associated with early glaucomatous changes. Invest Ophthalmol Vis Sci 2003;44(4):1586-90.

4. Lam DS, Leung DY, Chiu TY, et al. Pressure phosphene self-tonometry: a comparison with goldmann tonometry in glaucoma patients. Invest Ophthalmol Vis Sci 2004;45(9):3131-6.

5. Tai MC, Chen PL, Wu JN, Lu DW. Clinical evaluation of the intraocular pressure in patients with glaucoma or ocular hypertension by a self-assessable tonometer. J Ocul Pharmacol Ther 2005;21(1):55-61.

6. Liang SY, Lee GA, Shields D. Self-tonometry in glaucoma management--past, present and future. Surv Ophthalmol 2009;54(4):450-62.

7. Downs JC, Burgoyne CF, Seigfreid WP, et al. 24-hour IOP telemetry in the nonhuman primate: implant system performance and initial characterization of IOP at multiple timescales. Invest Ophthalmol Vis Sci 2011;52(10):7365-75.

8. Ittoop SM, SooHoo JR, Seibold LK, et al. Systematic Review of Current Devices for 24-h Intraocular Pressure Monitoring. Adv Ther 2016;33(10):1679-90.

9. Koutsonas A, Walter P, Roessler G, Plange N. Implantation of a novel telemetric intraocular pressure sensor in patients with glaucoma (ARGOS study): 1-year results. Invest Ophthalmol Vis Sci 2015;56(2):1063-9.

10. Twa MD, Roberts CJ, Karol HJ, et al. Evaluation of a contact lens-embedded sensor for intraocular pressure measurement. J Glaucoma 2010;19(6):382-90.

11. Kingman S. Glaucoma is second leading cause of blindness globally. Bull World Health Organ 2004;82(11):887-8.

12. De Moraes CG, Juthani VJ, Liebmann JM, et al. Risk factors for visual field progression in treated glaucoma. Arch Ophthalmol 2011;129(5):562-8.

13. Tezel G, Li LY, Patil RV, Wax MB. TNF-alpha and TNF-alpha receptor-1 in the retina of normal and glaucomatous eyes. Invest Ophthalmol Vis Sci 2001;42(8):1787-94.

14. Burgoyne CF, Downs JC. Premise and prediction-how optic nerve head biomechanics underlies the susceptibility and clinical behavior of the aged optic nerve head. J Glaucoma 2008;17(4):318-28.

15. Tezel G, Wax MB. Hypoxia-inducible factor 1alpha in the glaucomatous retina and optic nerve head. Arch Ophthalmol 2004;122(9):1348-56.

16. Yang J, Yang P, Tezel G, et al. Induction of HLA-DR expression in human lamina cribrosa astrocytes by cytokines and simulated ischemia. Invest Ophthalmol Vis Sci 2001;42(2):365-71.

17. Streit WJ, Mrak RE, Griffin WS. Microglia and neuroinflammation: a pathological perspective. J Neuroinflammation 2004;1(1):14.

18. (EGS) EGS. Terminology and Guidelines for Glaucoma, 4th ed. EU: PubliComm, 2014.

19. Dielemans I, Vingerling JR, Wolfs RC, et al. The prevalence of primary open-angle glaucoma in a population-based study in The Netherlands. The Rotterdam Study. Ophthalmology 1994;101(11):1851-5.

20. Rudnicka AR, Mt-Isa S, Owen CG, et al. Variations in primary open-angle glaucoma prevalence by age, gender, and race: a Bayesian meta-analysis. Invest Ophthalmol Vis Sci 2006;47(10):4254-61.

21. Sommer A, Tielsch JM, Katz J, et al. Relationship between intraocular pressure and primary open angle glaucoma among white and black Americans. The Baltimore Eye Survey. Arch Ophthalmol 1991;109(8):1090-5.

22. Tielsch JM, Katz J, Singh K, et al. A population-based evaluation of glaucoma screening: the Baltimore Eye Survey. Am J Epidemiol 1991;134(10):1102-10.

23. Cho HK, Kee C. Population-based glaucoma prevalence studies in Asians. Surv Ophthalmol 2014;59(4):434-47.

24. Detry-Morel M. Currents on target intraocular pressure and intraocular pressure fluctuations in glaucoma management. Bull Soc Belge Ophtalmol 2008(308):35-43.

25. Flammer J, Gugleta K, Hauenstein-Stümpfig D, et al. Glaucoma, 3rd revised ed: Hogrefe & Huber, 2006.

26. Caprioli J, Coleman AL. Intraocular pressure fluctuation a risk factor for visual field progression at low intraocular pressures in the advanced glaucoma intervention study. Ophthalmology 2008;115(7):1123-9.

27. Fogagnolo P, Orzalesi N, Ferreras A, Rossetti L. The circadian curve of intraocular pressure: can we estimate its characteristics during office hours? Invest Ophthalmol Vis Sci 2009;50(5):2209-15.

28. Sit AJ. Continuous monitoring of intraocular pressure: rationale and progress toward a clinical device. J Glaucoma 2009;18(4):272-9.

29. Orzalesi N, Rossetti L, Invernizzi T, et al. Effect of timolol, latanoprost, and dorzolamide on circadian IOP in glaucoma or ocular hypertension. Invest Ophthalmol Vis Sci 2000;41(9):2566-73.

30. Tarkkanen A, Ulfves K, Ulfves T. Self-tonometry in glaucoma. Graefes Arch Clin Exp Ophthalmol 2010;248(11):1679-81.

31. Todani A, Behlau I, Fava MA, et al. Intraocular pressure measurement by radio wave telemetry. Invest Ophthalmol Vis Sci 2011;52(13):9573-80.

32. Mansouri K. The road ahead to continuous 24-hour intraocular pressure monitoring in glaucoma. J Ophthalmic Vis Res 2014;9(2):260-8.

33. Greene ME, Gilman BG. Intraocular pressure measurement with instrumented contact lenses. Invest Ophthalmol 1974;13(4):299-302.
